# Feeding the cosmos: tackling personalized space nutrition and the leaky gut challenge

**DOI:** 10.1038/s41526-025-00490-z

**Published:** 2025-07-18

**Authors:** B. Barbero Barcenilla, R. Rivero, A. Lynch, W. Cromer, J. Gong, B. Harandi, M. Stegmann, H. Le, D. Lundine, M. Chung, J. Puig, K. Mikhailova, H. Coker, A. Marks, R. Gilbert, R. Scott, R. Barker, P. Glowe, Eliah G. Overbey, C. E. Mason

**Affiliations:** 1 Department of Biochemistry and Biophysics, Texas A&M University, College Station, TX USA; 2 BioAstra, Los Angeles, CA USA; 3 Department of Molecular, Cellular, and Developmental Biology, University of Michigan, Ann Arbor, MI USA; 4 Graduate Program in Developmental Biology, Baylor College of Medicine, Houston, TX USA; 5 Department of Translational Molecular Pathology, The University of Texas MD Anderson Cancer Center, Houston, TX USA; 6 Texas A&M Health Science Center, Medical Physiology, Texas A&M University, College Station, TX USA; 7 School of Computing, University of Wyoming, Laramie, WY USA; 8 Case Western Reserve University, Cleveland, OH, USA; 9 Western University of Health Sciences, College of Veterinary Medicine, Pomona, CA USA; 10 Memorial Sloan Kettering Cancer Center, department of radiation oncology, New York, NY USA; 11 Sonrai Analytics, Belfast, UK; 12 Cornell University, Ithaca, NY USA; 13 Department of Soil and Crop Sciences, Texas A&M University, College Station, TX USA; 14 Purdue University, West Lafayette, IN USA; 15 Seabrook Solutions, Space Biosciences Division, NASA Ames Research Center, Moffett Field, CA USA; 16 Amentum, Space Biosciences Division, NASA Ames Research Center, Moffett Field, CA USA; 17 Agricultural and biological engineering department, Purdue University, West Lafayette, IN USA; 18 Center for STEM, University of Austin, Austin, TX USA; 19 Department of Physiology and Biophysics and Tri-Institutional Computational Biology and Medicine Program, Weill Cornell Medicine, New York, NY USA; 20 The HRH Prince Alwaleed Bin Talal Bin Abdulaziz Alsaud Institute for Computational Biomedicine, Weill Cornell Medicine, New York, NY USA; 21 The WorldQuant Initiative for Quantitative Prediction, New York, NY USA; 22 The Feil Family Brain and Mind Research Institute, Weill Cornell Medicine, NY USA

**Keywords:** Disease prevention, Nutrition, Plant sciences, Risk factors

## Abstract

Long-duration space missions pose serious challenges to astronaut nutrition and health due to the altered environment of Low Earth Orbit (LEO). This study examines the nutritional composition of crops grown in space, identifying deficiencies in key nutrients such as calcium and magnesium, along with variable antioxidant profiles. These imbalances may impact astronaut physiology, particularly bone health and immune function, and are potentially linked to altered gene expression pathways in microgravity. Emerging evidence also suggests increased intestinal permeability, referred as leaky gut syndrome, which further disrupts nutrient absorption and immune regulation. To mitigate these issues, we evaluate targeted strategies including bioengineering of nutrient-dense crops, incorporation of antioxidant-rich species, and personalized nutrition guided by pharmacogenomics. Approaches such as biofortification and tailored supplementation are proposed to address these challenges. This work contributes to the development of resilient space agriculture systems that support astronaut health during deep space missions and future planetary habitation.

## Introduction

As humanity progresses deeper into the cosmos, the imperative for sustainable food production systems and effective nutritional strategies becomes increasingly paramount^1^. Space agriculture, which entails the cultivation of crops in extraterrestrial settings, holds significant potential for facilitating prolonged missions and future extraterrestrial settlements^2^. Nevertheless, the distinctive conditions prevailing in space pose considerable challenges to preserving the nutritional integrity of food cultivated beyond Earth’s atmosphere and ensuring the optimal health of astronauts^3^.

Optimal nutrition is essential for astronaut health and performance throughout extended space missions, as the human organism experiences numerous physiological transformations in microgravity, encompassing bone and muscle degradation^4^, altered immune functionality^5^, and cardiovascular deconditioning^6^. In addressing the challenges of sustaining human life, ensuring both caloric sufficiency and nutritional adequacy is essential. In line with current nutritional science^7^, there is an established recognition that spaceflight induced nutrition must consider the synergistic effects of macro- and micronutrients, bioactivate compounds, and their influence on immunity, metabolism, and tissue repair. Diets optimized with biofunctional compounds—such as flavonoids, vitamins, and essential trace elements—are necessary as countermeasures against the cumulative stresses of spaceflight to mitigate risks of nutrient deficiencies and maintain long-term astronaut health. In low Earth orbit (LEO), astronauts often experience weight loss and prepacked food quality declines over time^8^. Nevertheless, food cultivated in extraterrestrial environments encounters various impediments that can detrimentally influence its nutritional value. Cosmic radiation has the capacity to induce alterations in plant metabolic processes and nutrient composition, potentially culminating in diminished nutritional quality^9–11^. The lack of gravitational force further affects plant growth and development by altering nutrient uptake, gas exchange, and photosynthesis within higher plants^12,13^. Furthermore, limitations concerning water, nutrients, and cultivation volume within extraterrestrial habitats can adversely affect crop yield and nutritional composition^14^.

Low Earth Orbit stations, highlighting Tiangong-II and the International Space Station (ISS), have emerged as instrumental platforms for executing plant growth experiments in microgravity. Investigations such as lettuce grown on Veggie^15^ have substantiated the feasibility of cultivating crops in space and have offered insights into the challenges and prospective solutions pertinent to space agriculture. LEO-grown lettuce demonstrated differences in Ca, Fe, K, Na, P, S and Zn with all elements but Fe being reduced in the space-grown lettuce, which possibly may lead to deficiencies in meeting the daily nutritional requirements for human consumption^15^. This discrepancy and variability among plants raises concerns about the long-term sustainability of relying solely on space-grown crops for astronaut diets during extended missions on low Earth orbit.

Recent investigations, including the groundbreaking JAXA CFE study, NASA’s Twins Study^16^, and the I4 mission and Space Omics and Medical Atlas (SOMA)^17^, have produced a wealth of invaluable insights into the complex molecular and physiological responses triggered by the unique environment of spaceflight^18^. These studies have highlighted significant alterations in various biological systems, shedding light on how prolonged exposure to microgravity can impact human health at the cellular level. The findings underscore the critical importance of implementing targeted nutritional interventions to mitigate these effects and promote astronaut well-being during extended missions, especially if food source will be relied on crops grown aboard such stations.

Calcium has been prominently emphasized and recognized as one critical limiting factor that affect both the growth of plants cultivated in the unique environment of the International Space Station (ISS) and the overall health and well-being of the astronauts who inhabit that extraordinary setting^19^. There is evidence that Ca^2+^ signaling may be ‘gravidependent’ as Ca channels are loaded more heavily in spaceflight compared to earth^20,21^. Calcium deficiency will lead to a range of issues, including compromised plant development^22^ and increased risk of bone density loss in astronauts^23^, highlighting the need for effective strategies to ensure adequate nutrient supply during space missions.

This manuscript endeavors to deliver a thorough analysis of the nutritional challenges associated with food cultivated in LEO. We re-analyze the impact of the space environment on crop nutrient composition, delve into the potential health ramifications for astronauts for relying on space-grown food, and examine the influence of genetic factors on nutrient utilization. By leveraging data from various astronaut missions and other contemporary space-omics studies, and contemplating the intricate interplay between environmental determinants, genetic predispositions, and nutritional requirements, we aspire to contribute to the formulation of strategies aimed at ensuring optimal nutrition in extraterrestrial settings. Ultimately, this examination fortifies the health and welfare of space travelers during lengthy missions and prepares the way for lasting human habitation beyond our home planet.

## Result and discussion

### Nutritional content of low Earth orbit grown food

**Fig. 1:**
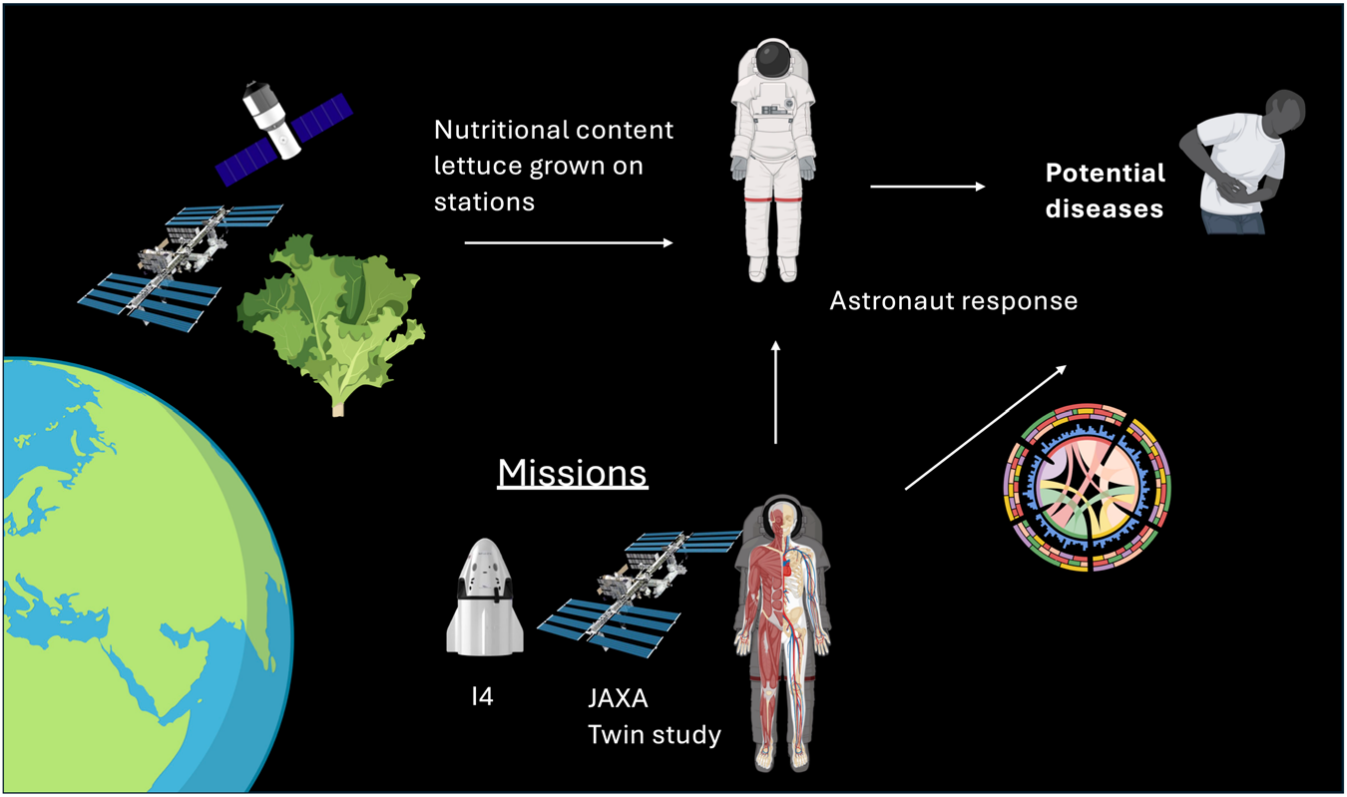
Conceptual framework of space-induced nutrient deficiencies and their potential impact on astronaut health. Representative model emphasizing the nutritional quality of crops grown in Low Earth Orbit (LEO) and the physiological effects of space-induced nutrient deficiencies on astronauts during various missions, which may be exacerbated by altered genetic expressions and potentially result in disease development.

**Fig. 2:**
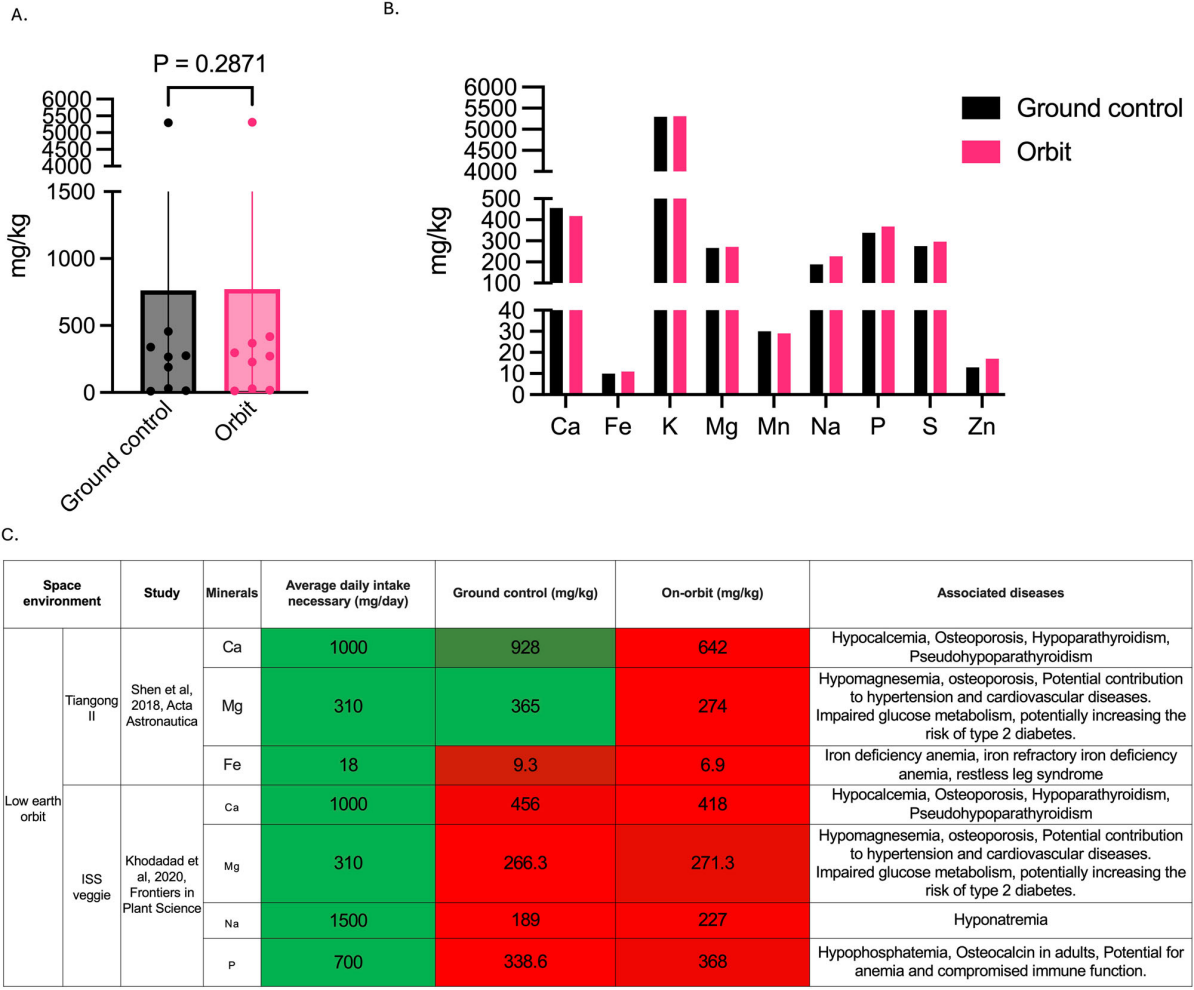
Nutritional dynamics of space-grown lettuce: mineral deficiencies, daily intake gaps, and health risks. **A** Nutritional content of ground control and space-flown lettuce, as analyzed by Shen et al.^25^ and Khodadad et al.^15^, without distinguishing between specific mineral or vitamin types. **B** Nutritional analysis categorized by mineral type. **C** A summary table highlighting the most relevant minerals based on average daily intake, comparing differences between ground control and space-flown content, and identifying diseases associated with deficiencies.

For long-term space missions, such as those to Mars or beyond, it is crucial to bring an adequate supply of food that meets astronauts’ nutritional needs (Fig. 1, Supplementary Table 1). While many different plants and crops have been grown in LEO (Supplementary Table 2)^24^, few experiments have focused on nutritional content of those plants compared to their ground control, ultimately limiting deep knowledge of species-specific interactions that occur due to growth in LEO. Studies conducted on the Tiangong II space station^25^ and the International Space Station (ISS) Veggie system^15,26,27^ provide valuable insights into the nutritional content of space-grown food compared to their Earth-grown counterparts. Overall, there was no change in nutritional content between ground control and LEO-grown lettuce aboard the different stations for lettuce when all nutrients were considered (Fig. 2a), despite the variability between different biological replicates^15^. In the Tiangong II study^25^, space-grown crops showed higher levels of potassium (K) compared to ground controls, with average 5840 mg kg^−1^ versus 5280 mg kg^−1^ respectively (Fig. 2b). An increase in K content of plants tissues is beneficial as K in humans plays a crucial role in maintaining proper fluid balance, nerve signals, and muscle contractions^28^. However, the same study revealed lower concentrations of Ca, Mg, and Fe in space-grown crops (Fig. 2b, c). Calcium levels decreased from 928 mg kg^−1^ to 642 mg kg^−1^, Mg from 365 mg kg^−1^ to 274 mg kg^−1^, and Fe from 9.3 mg kg^−1^ to 6.89 mg kg^−1^ (Fig. 2b, c). The ISS Veggie study showed similar trends but with some variations^15^. Potassium levels remained relatively stable between Earth and space-grown crops (5295 mg kg^−1^ vs. 5311 mg kg^−1^). Calcium showed a decrease in space (456 mg kg^−1^ to 418 mg kg^−1^), while Fe increased (10.33 mg kg^−1^ to 11.33 mg kg^−1^). Interestingly, this study also revealed increases in zinc (Zn) and phosphorus (P) content in space-grown crops.

When comparing the nutrition content of plants to the average daily intake requirements of humans, several key nutritional deficiencies become apparent. Calcium levels in space-grown crops fall significantly short of the recommended 1000–1300 mg day^−1^ intake (Fig. 2b, c)^29^. Magnesium levels are also below the recommended 310–420 mg day^−1^ (Fig. 2b, c)^30^. While Fe content in space-grown crops appears sufficient, the bioavailability of Fe in plant sources is generally lower than in animal sources, which could still lead to potential deficiencies^31,32^. These findings highlight the need for careful nutritional planning and potential supplementation strategies for long-term space missions relying on LEO-grown food sources to avoid nutrition-related diseases appearing on future missions.

### Antioxidant metabolites in space-grown food: assessing phenolics, ORAC, anthocyanins and carotenoids

In addition to the comprehensive nutritional analysis of certain vitamins and nutrients, researchers have also focused on measuring specific metabolites and antioxidants in space-grown crops. Of particular interest are phenolic compounds, oxygen radical absorbance capacity (ORAC), anthocyanins and carotenoids, which play crucial roles in the nutritional quality and antioxidant properties of plants. Analyzing data from Veggie 1, 2, and 3 experiments reveals that LEO-grown lettuce led to reduced total phenolics, while anthocyanins and antioxidant capacity (measured by ORAC) were not different from their ground controls^15^.

Anthocyanin levels remained relatively stable between space and ground samples. The recommended intake for anthocyanins is ~12.5 mg day^−1^ ^33^, and the space-grown lettuce consistently provided 3–5 μg mg^−1^ (Supplementary Fig. 1). Adequate intake of these compounds is crucial for preventing various health issues, including cardiovascular diseases, type 2 diabetes, liver diseases, osteoporosis, and age-related macular degeneration^34^. The antioxidant properties of these compounds also play a vital role in combating oxidative stress, which is of particular concern in the space environment.

In Veggie 2^15^, total phenolics content on station grown lettuce was a 0.1 mg g^−1^ compared to 49.6 mg g^−1^ for ground control (Supplementary Fig. 1a). While polyphenols offer numerous health benefits, establishing precise daily intake recommendations remains challenging due to variations in food content and individual absorption rates^35^. However, this experiment reveals that the phenol content of space-grown lettuce falls significantly below the average American intake of 450 mg day^−1^ ^36^ (Supplementary Fig. 1b). Phenol deficiency is concerning as inadequate polyphenol consumption has been associated with increased risks of cardiovascular diseases, diabetes mellitus, and cancer^37^. Nevertheless, in Veggie 1, space-grown lettuce showed higher levels of total phenolics (63.4 mg g^−1^) compared to the ground control (54.4 mg g^−1^), indicating a potential stress response to the space environment (Supplementary Fig. 1a).

Beyond the analysis revealed by Khodadad et al.^15^, analysis of secondary metabolites in ISS-grown plants revealed significant changes compared to Earth-grown controls by Raman spectroscopy^38^. This technique showed a substantial increase in the intensity of the phenylpropanoid peak at 1608 cm^−1^ for ISS-grown *Arabidopsis thaliana* compared to Earth-grown plants. Phenylpropanoid increase indicates a higher concentration of low molecular weight phenylpropanoids in space-grown plants, suggesting that ISS-grown plants experience spaceflight-induced stress not observed in Earth-grown plants. The study also observed a decrease in the intensity of vibrational bands associated with carotenoids (1115, 1155, 1186, 1215, and 1525 cm^−1^) in ISS-grown plants. This decrease suggests that plants in space may be activating enzymatic degradation of carotenoids to produce abscisic acid (ABA), a stress response hormone. Additionally, the reduction in carotenoids could be due to oxidation and fragmentation by reactive oxygen species (ROS), leading to the formation of β-lonone and β-cyclocitrals, which activate plant defense mechanisms. These findings demonstrate that the space environment induces significant changes in the secondary metabolite profile of plants, particularly in compounds related to stress responses.

The plant stress response phenotype was also observed in the ORAC values, with space-grown lettuce exhibiting higher antioxidant capacity (1063 μmol TE g^−1^) than the ground control (826.6 μmol TE g^−1^) (Supplementary Fig. 1)^15^. The observed data aligns with increased oxidative stress in space-grown plant genomes^39^. This elevated production of ROS in plants has prompted concerns about potential toxicity^40,41^ when incorporating these plants into astronaut diets. A major question is whether the heightened oxidative stress in space-grown plants has implications for both plant health and the nutritional quality of crops^40,42^.

### Health risks associated with nutritional deficiencies from space-grown crops

The combination of nutritional deficiencies and elevated ROS in space-grown plants consumed by astronauts may present substantial health risks during extended space missions^41^. These factors could potentially trigger a variety of diseases and disorders, jeopardizing both mission success and astronaut health (Fig. 1).

Calcium deficiency, a particular concern in space-grown crops, can lead to hypocalcemia, osteoporosis, and increased fracture risk (Fig. 2c, Supplementary Table 3)^23^. This is especially problematic given the bone loss already associated with microgravity exposure, and the presence of kidney stones in astronauts^43,44^. Iron deficiency, another nutrient often found in lower concentrations in space-grown food (Fig. 2c, Supplementary Table 3), may result in anemia, potentially affecting oxygen transport and astronaut performance^31^. Magnesium deficiency, also observed in space crops, can contribute to muscle cramps, osteoporosis, and hypertension. Vitamin D deficiency, exacerbated by lack of sunlight exposure in space, may lead to further bone health issues such as rickets or osteomalacia^45,46^. Additionally, deficiencies in antioxidants like vitamin C and E could weaken the immune system and increase susceptibility to radiation damage^47^, while inadequate vitamin B12 intake might result in neurological disorders and pernicious anemia^48^. Furthermore, the combined effects of multiple nutrient deficiencies could synergistically impact astronaut health, potentially leading to complex, difficult-to-treat conditions.

### Genetic factors influencing nutrient utilization from space-grown crops in astronauts: a focus on calcium

The nutritional deficiencies in space-grown plants present significant health risks for astronauts during extended missions. These risks are further complicated by genetic factors and gene expression changes induced by the space environment. The individual biochemistry of each astronaut adds another layer of complexity to addressing these nutritional challenges effectively. A primary concern is the potential insufficient Ca content in crops grown in microgravity, which could exacerbate Ca-related health complications experienced by astronauts during long-duration missions^43,49,50^. Given this issue, we analyzed the biochemistry and expression patterns of calcium-related pathways in astronauts across various space missions.

**Fig. 3:**
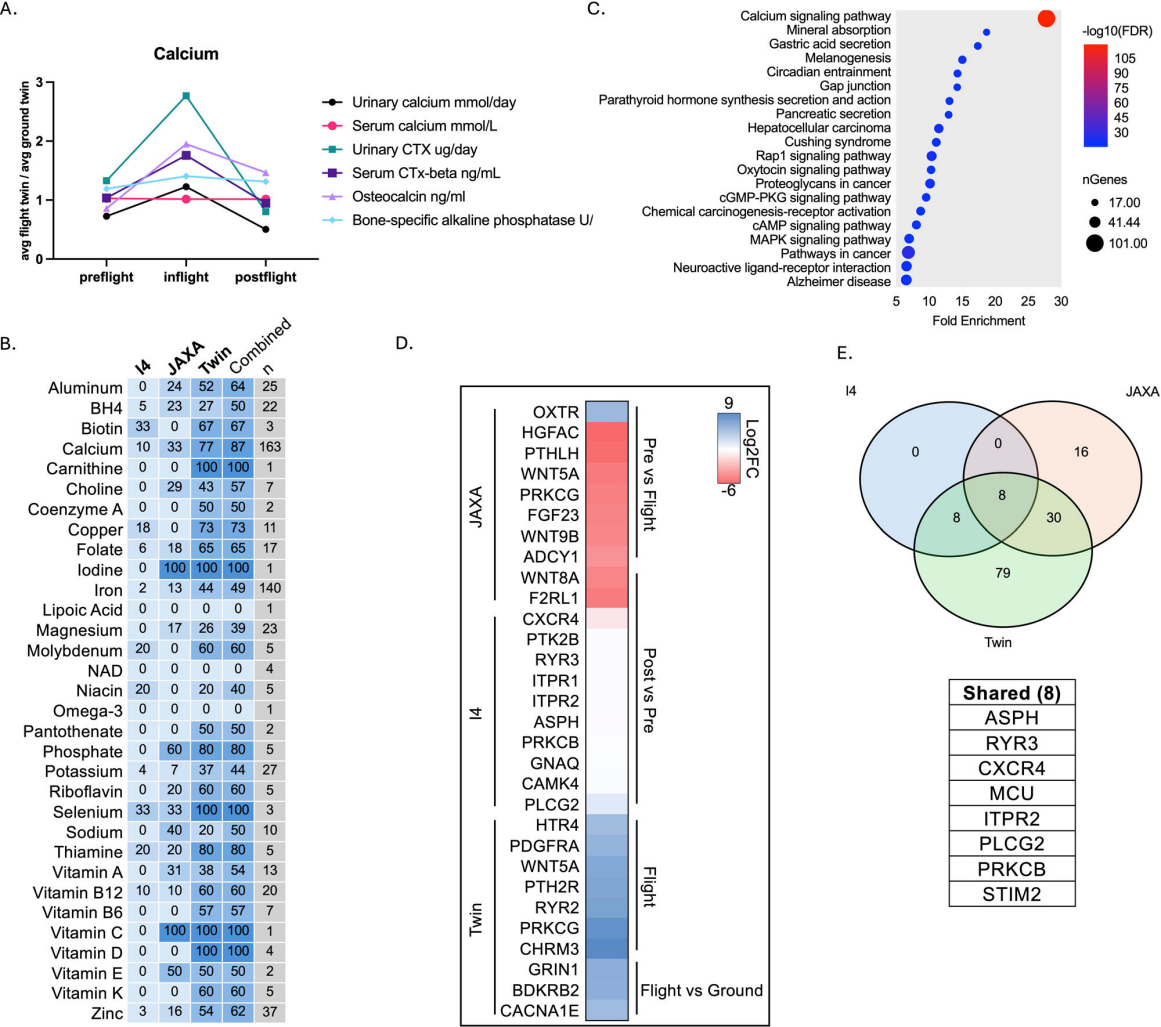
Nutritional and calcium-related changes in astronauts, irrespective of cell type, observed in the I4, Twin Study, and JAXA missions. **A** Biochemical analysis of calcium-related molecules for Twin-study mission. **B** Percentage of DEGs among queried gene sets related to different vitamins and nutrients for the I4, JAXA and Twin study missions. **C** KEGG pathway association of DEG from combined missions from nutrition-related genes selected. **D** Top 10 Ca-related DEGs according to Log2FC irrespective of cell type for each study (*p* < 0.05). *Log2FC* was averaged to combine cell types where applicable. **E** Venn diagram displaying all Calcium DEGs between study with a table outlining shared calcium-related genes among missions.

Supporting the hypothesis of deficient Ca in both crops and astronauts, biochemical data from twin study and I4 missions indicate an increase in urinary Ca and osteocalcin levels during spaceflight^16,51–53^ (Fig. 3a). Urinary Ca increased significantly during the inflight period, rising from 0.73 mmol day^−1^ preflight to 1.23 mmol day^−1^ inflight, before decreasing to 0.50 mmol day^−1^ postflight. Serum Ca remained relatively stable across all phases. Urinary CTX showed a substantial increase from 1.33 μg day^−1^ preflight to 2.77 μg day^−1^ inflight, followed by a decrease to 0.80 μg day postflight^−1^. Serum CTx-beta levels also increased inflight (1.76 ng mL^−1^) compared to preflight (1.04 ng mL^−1^) and postflight (0.95 ng mL^−1^). Osteocalcin levels rose from 0.86 ng mL^−1^ preflight to 1.95 ng mL^−1^ inflight, then decreased to 1.47 ng mL^−1^ postflight. Bone-specific alkaline phosphatase showed a modest increase from 1.19 U L^−1^ preflight to 1.41 U L^−1^ inflight, with a slight decrease to 1.31 U L^−1^ postflight. These changes suggest increased bone turnover during the inflight period, with markers generally returning towards baseline levels postflight. Calcium changes may be explained by the significant differential expression of Ca-related genes compared to other nutrient-related pathways (Fig. 3b, Supplementary Table 4).

Looking at the different mission profiles including Twin study experiment, Inspiration 4 and JAXA, Ca signaling pathway was heavily enriched for nutrient-related genes that are differentially expressed for preflight compared to flight or postflight (Fig. 3c, Supplementary Table 4). Among the 163 different Ca genes looked for the different missions (Fig. 3b), 8 were shared among them (Fig. 3d, e), highlighting ASPH, RYR3, CXCR4, MCU, ITPR2, PLCG2, PRKCB and STIM2. Misregulation of these genes could lead to hypocalcemia, osteoporosis, hypoparathyroidism, or pseudohypoparathyroidism, potentially worsening the effects of Ca deficiency in space-grown foods^54,55^.

Considering individual mission profiles, genes significantly downregulated (padj. <0.05) in the JAXA data suggest modulation of Ca pathways, likely playing a role in bone health changes (Fig. 3D). *WNT5A* is part of the Wnt signaling pathway, crucial for bone formation and regeneration^56^. WNT5A specifically contributes to osteoblast differentiation, which is key to building bone tissue. Downregulation can disrupt these pathways, leading to weaker bone structure and a higher risk of fractures^57^. *PTHLH* (Parathyroid Hormone-Like Hormone) helps regulate Ca levels in bones and maintains bone density through paracrine signaling, impacting osteoclast and osteoblast activity^58^. Downregulation of PTHLH could lead to bone resorption exceeding bone formation, resulting in bone loss. *NTRK2* and *GRM1* are involved in neural and signaling pathways that may indirectly impact bone density by influencing bone cell behavior through neurotransmitter signaling (Supplementary Table 4)^59–61^. *ADCY1* is involved in cellular signaling that affects bone cell activity. *ADCY1* is implicated in bone health, while the broader family of adenylate cyclases and their involvement in the cAMP signaling pathway are established regulators of bone metabolism^62,63^. Also, fibroblast growth factor 23 (FGF23) is crucial for skeletal development and bone mineralization because it regulates phosphate and vitamin D levels with excess FGF23 causing hypophosphatemia, leading to impaired bone mineralization and results in skeletal defects^64^.

### Cellular diversity response for calcium-related genes in different space missions

**Fig. 4:**
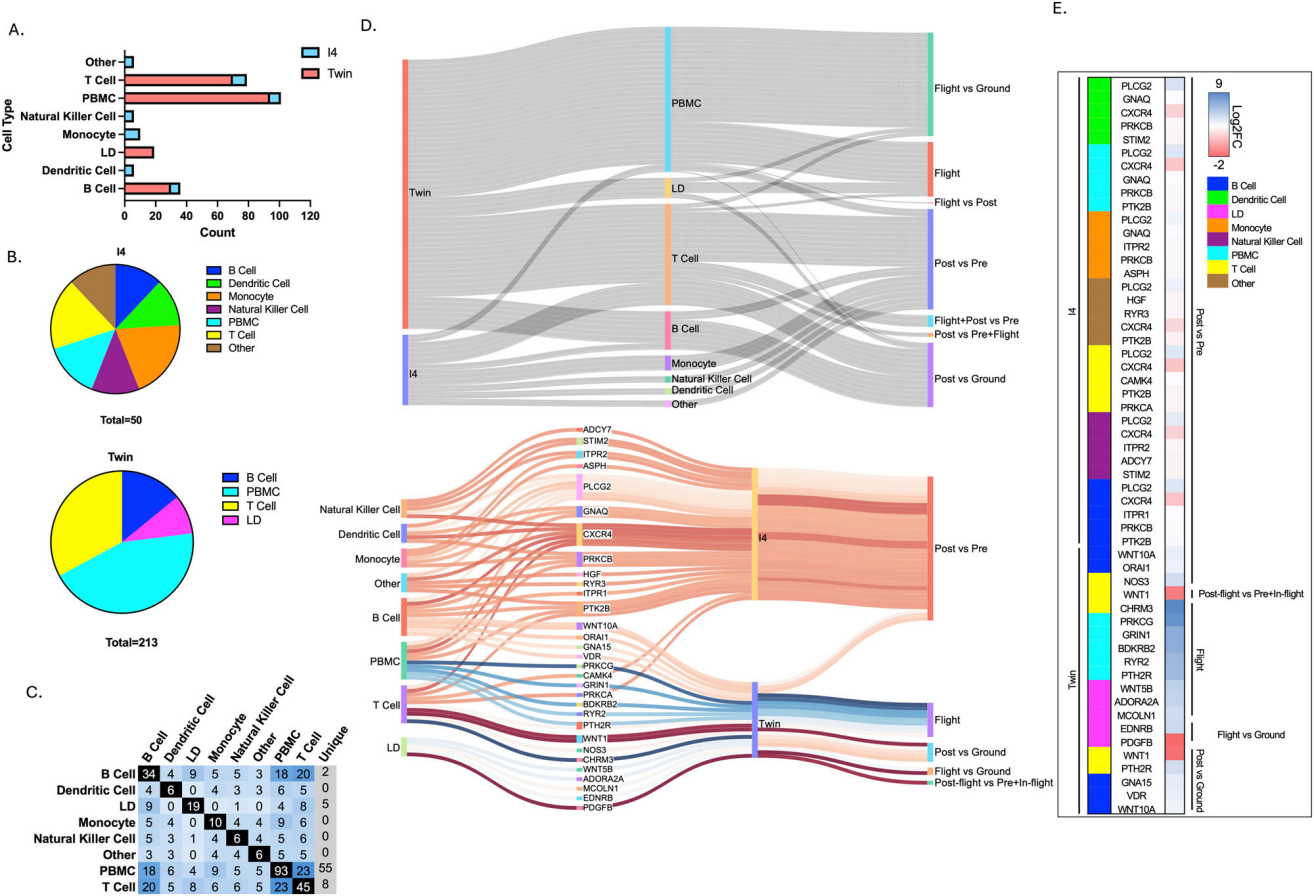
Analysis of Calcium-related Differentially Expressed Genes (DEGs) across I4 and twin study missions by cell type. **A** Calcium-related DEG count for I4 and Twin study by cell type. **B** Venn diagram of I4 mission (top) and Twin study (bottom) cell counts. **C** Pairwise comparison of overlapping Ca-related DEG counts between cell types for combined I4 and Twin study missions. **D** (Top) All Calcium-related DEG-cell type -contrast associations (Clustered cell types and contrasts). (bottom) Top 5 calcium-related DEGs for each cell type per study with Log2FC represented by line color (red = minimum/blue = maximum). **E** Top 5 Calcium-related DEGs for each cell type per study.

Analyzing different cell types in astronauts is crucial for understanding the comprehensive impact of spaceflight on human physiology and nutrition. The space environment elicits unique responses from various cell types, particularly in Ca-related pathways. Gene expression data reveals distinct patterns across CD4 T cells, CD8 T cells, B cells (CD19), and lymphoid cells (LD) in their Ca responses (Fig. 4). While different space missions consistently highlight the importance of Ca signaling, the specific genes affected in each study suggest distinct aspects of Ca health regulation.

DE of Ca-related genes appears in the I4 data, particularly noteworthy in the CD14+ monocyte cell-type due to their role as precursor cells to osteoclasts that resorb bone tissue (Fig. 4a, b). Half of the significant Ca-related I4 DEGs overlap with DEGs in the JAXA data and are involved in similar pathways with slight upregulation (log2FC < 1) compared to higher downregulation of JAXA data (log2FC > 1) (Fig. 4c, d) (Supplementary Table 4). *PLCG2* is a signaling enzyme in osteoclast differentiation, similar to *WNT5A* in JAXA data. Both of these genes influence RANKL signaling and osteoclast activity which impact bone density through NF-KB and NFAT pathways^65,66^. *PRKCB* links with intracellular signaling like *PRKCG*. *PRKCB* modulates bone turnover and cellular responses in osteoclast activity. *GNAQ* involved in Ca signaling indirectly supports WNT and *PRKCG* pathways relating to Ca homeostasis in the bone. In addition to *ACDY1*, it regulates intracellular Ca in osteoclast differentiation and bone maintenance^66^. *ASPH* may overlap with *PTHLH* in cell attachment and differentiation (not well characterized in CD14+ monocytes). *ITPR2* works in Ca release and complements *FGF23* in nutrient homeostasis^65^. *PTK2B* influences osteoclast attachment to bone, similarly to *PRKCG*. *PTK2B* and *WNT5A* work together in cytoskeletal organization and impact osteoclast migration involved in bone resorption and remodeling (Fig. 4d, e).

In the Twin study, significant DE of Ca-related genes were observed in CD4 cells and LD cells, offering insights into the physiological responses to spaceflight (Fig. 4a, b). CD4 T cells play a role in Ca-related bone health through their regulation of bone remodeling. Th17 cells enhance osteoclast activity, promoting bone resorption and Ca release, while Tregs counteract this by suppressing osteoclastogenesis and inflammation^67^. Dendritic cells (e.g., LD cells) influence bone health indirectly by shaping CD4 T cell responses^68^. Chronic immune activation involving these cells is associated with bone loss in conditions like osteoporosis^69^.

In the Twin study, upregulated genes in CD4 cells include *PTH2R*, *ADCY2*, and *CACNA1A*, all of which play roles in calcium signaling (Fig. 4e). *PTH2R*, a receptor for parathyroid hormone, modulates Ca homeostasis and may influence immune cell activity in space by regulating intracellular calcium levels. *ADCY2*, part of the adenylate cyclase family, contributes to cAMP production, which is vital for Ca-mediated signaling pathways that affect T-cell activation and differentiation^70^. *CACNA1A*, while primarily known for its role in neurons, encodes a subunit of a voltage-dependent Ca channel and is shown in the data to be expressed in T cells, perhaps impacting their signaling^71^.

Conversely, downregulated genes include *ADRB2*, *VDR*, and *CACNA1H* (Fig. 4e). *ADRB2* encodes the beta-2 adrenergic receptor, which impacts intracellular Ca flux and immune cell migration, potentially affecting CD4 cell responsiveness^72,73^. *VDR*, the vitamin D receptor, is critical for Ca absorption and immune modulation, and its downregulation could impair T-cell responses and contribute to reduced Ca availability in space conditions^74^. *CACNA1H*, downregulated in CD4 cells, encodes a subunit of voltage-gated Ca channels that influence Ca signaling in osteoclasts, potentially contributing to bone loss^75,76^.

Notably, there are overlaps with findings from the JAXA study and in general pathways from I4, emphasizing shared pathways affected by spaceflight (Supplementary Fig. 2). *ADCY2* aligns with the upregulation of *ADCY1* observed in the JAXA data, highlighting the role of adenylate cyclases in Ca signaling and bone cell activity. Similarly, *VDR* complements the downregulation of *FGF23* and *PTHLH* in JAXA data, linking to disrupted Ca-phosphate homeostasis. These results underscore the interconnected impact of Ca signaling genes across immune and bone-related pathways during and after spaceflight, reinforcing the critical need to monitor and mitigate Ca-related physiological changes in astronauts^77^.

In addition to CD14 cells and CD4 cells, examining B cells in astronauts is crucial for understanding the impact of spaceflight on the immune system. B cells play a vital role in adaptive immunity by producing antibodies, presenting antigens, and regulating immune responses. We found a significant change in expression on Ca related genes in B cells from I4 astronauts (Supplementary Table 5). This alteration in B cell activity may lead to reduced vaccine efficacy, increased susceptibility to infections, and potential long-term health risks for astronauts during extended missions. Key genes such as PLCG2, ITPR1, PTK2B, and PRKCB, which are essential for B cell activation and function, show altered expression patterns. PLCG2 encodes for phospholipase C gamma 2, a key enzyme in the B cell receptor (BCR) signaling cascade, while ITPR1 encodes for inositol trisphosphate receptor type 1, which is vital for Ca release from the endoplasmic reticulum. The dysregulation of genes encoding inositol trisphosphate receptors (IP3Rs) and transient receptor potential (TRP) channels is particularly concerning, as these are critical for Ca entry and release in B cells. Such gene alterations, combined with low Ca generated by space-grown crops, could contribute to the immune dysfunction observed in astronauts during space missions, potentially increasing their susceptibility to infections and reducing vaccine effectiveness.

### Calcium changes in mitochondria related genes

Recent studies examining astronaut gene expression while in space have revealed notable changes in genes associated with mitochondria^78–80^. The varying expression of these genes might indicate the body’s effort to manage heightened oxidative stress and metabolic requirements during space travel; nevertheless, numerous mitochondrial-related genes are essential for nutrient metabolism. Calcium plays a crucial role in mitochondrial function, which is particularly significant for astronauts during extended space missions. Calcium ions act as important signaling molecules within mitochondria, influencing various metabolic processes, including ATP synthesis and the regulation of reactive oxygen species ROS.

Notably, genes involved in Ca signaling pathways, such as SMDT1 and PRDX5, show significant downregulation during spaceflight (Supplementary Table 6). PRDX5 shows the highest level of down regulation with a logFoldChange of −3.616 among the investigated genes. PRDX5 influences Ca uptake into mitochondria by regulating the mitochondrial redox state, which is essential for cellular function and neuronal survival. This is particularly relevant for neurodegenerative diseases such as Parkinson’s, as mitochondrial stress and disturbed calcium dynamics are crucial mechanisms in the pathogenesis of these diseases^81^. The altered expression of genes involved in detoxification processes, such as NDUFAB1, MPST, and ACSM5, further emphasizes the need for carefully tailored nutritional strategies to support mitochondrial function and overall health in the unique space environment. NDUFAB1 is an essential mitochondrial acyl carrier protein and cofactor for complex I in the respiratory chain and takes part at the Fe-S cluster biosynthesis and detoxification^82,83^.

While it does not directly regulate Ca-induced ATP synthesis, it plays a vital role in maintaining the stability and functionality of complex I. Increased Ca influx can enhance complex I activity, which subsequently leads to the production of ROS. Defects in complex I components, including NDUFAB1, are implicated in various mitochondrial diseases. Studies have shown that cardiac deletion of NDUFAB1 leads to impaired bioenergetics and increased ROS levels, resulting in progressive dilated cardiomyopathy and increased mortality due to heart failure^84^. Furthermore, it has the potential to mitigate the risk of obesity or insulin resistance^85^.

### Calcium signaling pathway variations across different space missions

Calcium signaling plays a crucial role in various cellular processes, such as metabolism, signal transduction, and cell survival, all of which are notably affected across different cell types and mission. Gaining an understanding of how these pathways adapt or alter during spaceflight could offer valuable insights into the physiological challenges astronauts face with different mission profiles. By analyzing the Ca DEG through KEGG, a comprehensive comparison of changes in Ca signaling pathways can be made (Supplementary Fig. 2).

The shorter I4 mission underscores early intracellular responses to spaceflight-induced stress, with a specific focus on endoplasmic reticulum (ER)-mediated Ca regulation via IP3R and RYR channels (Supplementary Fig. 2a, left). These mechanisms primarily impair osteoblast activity while sparing mitochondrial involvement, suggesting a lesser role of oxidative stress in this context. The minimal extracellular Ca influx through voltage-gated Ca channels (VGCCs) reflects a contained, localized stress response. These findings suggest that early or mild stress primarily affects bone formation through intracellular signaling, with limited engagement of broader oxidative or transcriptional stress pathways.

The JAXA mission, in contrast, reveals a broader extracellular Ca reliance through VGCCs, indicative of systemic stress affecting multiple cellular systems (Supplementary Fig. 2a, right). Mitochondrial involvement, including the mitochondrial Ca uniporter (MCU), suggests heightened oxidative stress and energy imbalances, contributing to osteoclast activation. Additionally, downstream transcriptional regulators such as NFAT point to a disruption of apoptosis and gene expression pathways, with significant implications for bone resorption and tissue remodeling. This wider Ca dysregulation highlights how spaceflight conditions escalate stress from localized to systemic, exacerbating oxidative damage and metabolic dysfunctions.

The long twin study mission presents the most severe impacts, with extensive disruptions in both extracellular and intracellular Ca regulation (Supplementary Fig. 2a, right). Heavy mitochondrial involvement drives severe oxidative stress, energy imbalances, and impaired metabolic and transcriptional regulation. Key pathways, including NFAT and CREB, underline the compounded effects on bone, muscle, immune, and cognitive systems, pointing to a holistic stress response. Shared features across the studies, such as mitochondrial Ca dysregulation and ER stress, highlight universal pathways disrupted by spaceflight. These findings link NFAT and PKC dysregulation to bone health, CaMKII dysfunction to muscle atrophy, and mitochondrial ROS to oxidative stress and cell death.

The major changes across the studies highlight varying degrees of Ca dysregulation and stress responses. The I4 study focuses on early intracellular effects, with ER-specific Ca dysregulation (IP3R/RYR) impairing osteoblast activity and minimal mitochondrial involvement, suggesting limited oxidative stress. The JAXA study reveals extracellular Ca influx (VGCCs) and downstream mitochondrial activity, indicating heightened oxidative stress, osteoclast activation, and systematic transcriptional regulation (NFAT). The twin study shows the most severe impacts, with widespread disruptions in both extracellular and intracellular Ca regulation, heavy mitochondrial involvement, and severe oxidative stress causing metabolic and transcriptional imbalances. Despite these differences, all studies consistently point to shared mechanisms, including intracellular Ca release through IP3R/RYR, mitochondrial stress (MCU), and transcriptional dysregulation (NFAT/CREB), which are central to the disruption of bone, muscle, immune, and cognitive health. These findings underscore the fundamental pathways affected by spaceflight, linking Ca dysregulation to oxidative stress, tissue remodeling, and energy imbalances.

### Exploring genetic variations in nutrition and pharmacogenomics for astronaut health in space

Prior analysis has revealed specific genes associated with nutrition that demand further examination to ensure the capacity of astronauts to effectively digest food cultivated in extraterrestrial environments and to mitigate nutrition-related health issues. Through the application of pharmacogenomics, a field that explores how genetic variability affects pharmacological responses, several of the aforementioned genes may acquire heightened significance in pre-launch analyses, given that certain pharmaceuticals intended for space-related ailments might exhibit diminished efficacy in the presence of single nucleotide polymorphisms (SNPs) and gene variants. Such genetic variations could additionally affect the body’s capacity to metabolize and utilize nutrients, thereby highlighting the critical necessity of sustaining sufficient nutritional intake. The variants of nutrient-associated genes identified within the I4 dataset imply potential correlations among genetic predispositions, nutrient metabolism, and the inherent challenges of space travel (Supplementary Fig. 3).

“Nutritional pharmacogenomics” (Supplementary Table 7) highlights significant trends in the relationships between diseases, nutrients, drugs, and genetic variants, underscoring the complex interplay of these factors in health outcomes. Among the diseases listed associated with space-related variants, cancer is the most prevalent, with 47 associations, followed by hypertension with 13 (Supplementary Fig. 3, Supplementary Table 7). Cancer’s prominence reflects its intricate links to genetic predispositions, nutrient metabolism, and therapeutic interventions. Other conditions, though less frequent, provide additional insights into broader health challenges, including cardiovascular and neurological disorders.

Calcium and folate related genes are the most frequent, with 28 associations each. Genetic variants, such as those in *VDR*, *CACNA1A*, and *ADRB2*, may impair Ca absorption and utilization, compounding risks of bone demineralization and hypertension. Similarly, folate and vitamin-related genes like *MTHFR* and *SLC19A1* are vital for DNA repair, cell division, and oxidative damage prevention, making their variants particularly significant in space, where radiation can increase mutational risks. Trace mineral deficiencies also pose significant risks. Variants in genes like SLC31A1 (copper transport) and ADRB2 (linked to cardiovascular regulation) highlight the importance of Cu and Mg for combating oxidative stress and maintaining cardiovascular function. Vitamin K, linked to VKORC1 variants, is another critical vitamin that, when deficient, may increase thrombotic risks, emphasizing the importance of maintaining balanced nutrition. Cancer-related variants in genes like *VEGFA* and *ERBB3* are associated with oxidative stress and tumorigenesis, and deficiencies in folate, vitamin B12, or other antioxidants can further exacerbate these risks. Vitamin B12, essential for neurological health and anemia prevention, works synergistically with folate. A lack of these nutrients may worsen the predispositions to conditions such as schizophrenia, cardiovascular disease, and cancers. In addition, schizophrenia-related variants in *NTRK2* and *HTR7* further emphasize the critical role of mental health support in space.

Collectively, the findings reveal a strong connection between genetic predispositions and nutrient metabolism, particularly in the context of complex diseases like cancer, cardiovascular disorders, and mental health conditions. Variants such as rs17421511, rs3025040, and rs1801131 recur frequently, suggesting their significant roles in drug responses and disease mechanisms. The interconnected nature of these factors underscores the need for targeted medical research and personalized healthcare strategies, particularly in extreme environments like space, where environmental stressors can amplify genetic vulnerabilities.

### Crop selection, nutrient supplementation and plant bioengineering to address calcium deficiencies in space

**Fig. 5:**
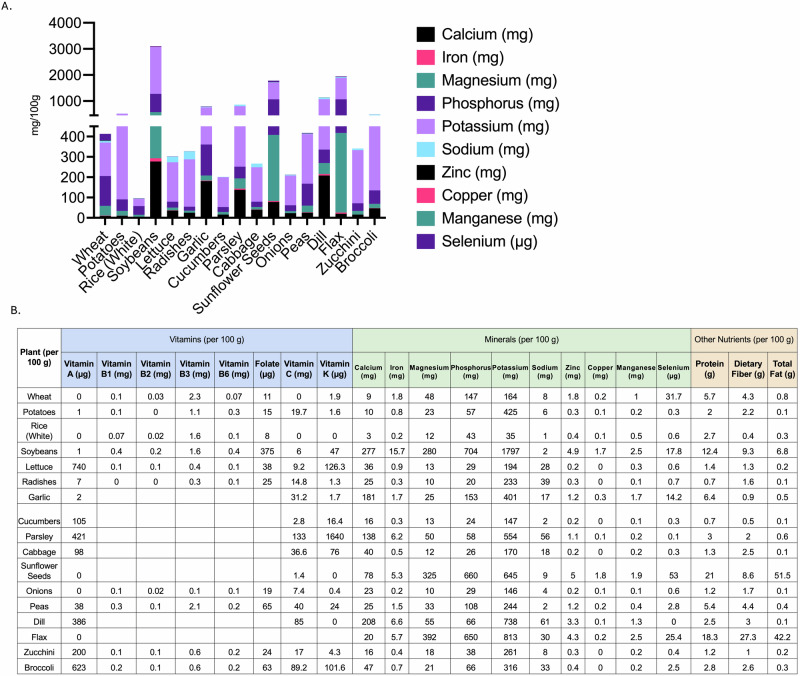
Nutritional profile of space-relevant crops. **A** Graph displaying mineral content of different space-relevant crops in mg/100 g. **B** Table displaying vitamins, minerals and other nutrients for space-relevant crops.

Previous analysis has revealed alterations in Ca signaling pathways in astronauts during various space missions, independent of the low Ca content in space-grown lettuce. Beyond lettuce^86^, there are several plants known for their high Ca content that are being considered for integration into space agriculture programs to supplement astronauts’ diets and mitigate the risks associated with prolonged space travel (Fig. 5a, b)^87,88^. Among the most promising candidates are soybeans, parsley, garlic, and flax. These plants not only offer substantial amounts of Ca but have also demonstrated adaptability to space environments in previous experiments. Soybeans, for instance, contain an impressive 2077 mg Ca kg^−1^ and have been successfully grown on the International Space Station (Fig. 5a, b, Supplementary Table 2). Parsley and garlic, with 1380 mg and 1810 mg Ca kg^−1^ respectively, have shown potential in various space station experiments (Fig. 5a, b, Supplementary Table 2). While leafy greens like lettuce and cabbage contain less Ca, they have proven to be practical choices for space cultivation due to their relatively quick growth cycles and adaptability to controlled environments.

Spaceflight induces significant physiological changes in astronauts, particularly affecting bone density and Ca metabolism. To address these physiological changes, we explored the potential of specific nutrients to modulate the Ca pathways associated with the previously discussed genes. Calcium pathways may be sensitive to modulation by certain nutrients, especially quercetin, vitamin D, and L-glutamic acid. Vitamin D enhances Wnt signaling, promoting bone formation by regulating Ca and P absorption, while balancing *FGF23*’s role in bone mineralization^89^. Quercetin, a flavonoid with anti-inflammatory properties, may inhibit osteoclast differentiation by targeting pathways like *PLCG2*, *PRKCB*, and *NF-kB*, reducing bone resorption and supporting Ca and P metabolism through Wnt signaling^90,91^. Furthermore, quercetin has been shown to complement *PTHLH*’s role in bone turnover by reducing osteoclastogenesis and modulating bone remodeling processes^92^. L-glutamic acid, a neurotransmitter, may stabilize pathways involving *GNAQ* and *PLCG2*, improving bone resorption and cellular signaling^93^. Together, these nutrients can synergistically support bone density, reduce excessive bone resorption, and maintain the balance between bone formation and turnover, highlighting their potential in bone health management to mitigate the harmful effects of spaceflight. By incorporating foods rich in these nutrients into space agriculture programs and astronaut diets, it may be possible to counteract some of the negative impacts of microgravity and radiation on Ca signaling and bone metabolism during long-duration space missions.

Alternative to nutrient supplementation strategies to mitigate the impacts of spaceflight-induced transcriptional changes, biofortification, the process of enhancing the nutritional profile of plants through genetic or agronomic practices, offers a sustainable solution to prevent nutrient deficiencies. As bone health is closely tied to nutrient availability, biofortifying plants like lettuce with key nutrients and vitamins—such as Ca, vitamin D, and Mg—could provide astronauts with a reliable food source that helps mitigate the effects of space-induced changes in bone metabolism. This method enhances the nutritional quality of produce, potentially reducing reliance on traditional supplements during extended missions and addressing gaps caused by limited food resources in space^94^. In addition to biofortification, plant-based systems have shown great promise for delivering therapeutic proteins that could address bone loss directly. Advancements in plant biotechnology have enabled the expression of therapeutic proteins, such as modified parathyroid hormone (PTH-Fc), in plants, offering innovative, plant-based solutions for managing osteoporosis and bone health challenges in astronauts^95^. PTH-Fc plays a crucial role in regulating Ca and P levels, enhancing bone formation while inhibiting excessive osteoclast activity. Plant-based expression of PTH-Fc could provide astronauts with a sustainable, easily administered treatment for osteoporosis and other bone-related issues that arise during prolonged space missions. This innovation not only offers an alternative to pharmaceutical treatments but also reduces the logistical challenges of transporting pharmaceutical drugs to space. Such an integrated approach addresses both the preventive and therapeutic aspects of bone health, reducing astronauts’ reliance on external supplies.

### Role of quercetin in mitigating space nutritional risks

Flavonoids, particularly quercetin, have emerged as promising compounds for space nutrition and astronaut health management. Space-based plant studies have consistently shown increased phenylpropanoid production, which includes flavonoids, in microgravity environments^38,96–98^. Quercetin stands out as a potent flavonoid antioxidant^99^. Its anti-inflammatory properties and ability to inhibit lipid peroxidation make it valuable for managing inflammatory disorders that may arise during long-duration missions^100^. As a powerful antioxidant, quercetin has been extensively studied for its potential in cancer therapy, which is relevant given the increased radiation exposure in space^101^. Moreover, quercetin’s protective effects against UV-induced damage and its anti-aging properties are particularly beneficial for maintaining skin health in the harsh space environment.

Genes such as *VEGFA*, found in the I4 variants dataset, are central to angiogenesis and oxidative stress regulation. Variants like rs3025040, rs833069, and rs13207351 are linked to cancer and associated with treatments such as cisplatin and carboplatin (Supplementary Fig. 3, Supplementary Table 7). Quercetin’s antioxidant activity, which scavenges free radicals and inhibits oxidative stress, aligns with its potential to mitigate oxidative damage driven by these variants. By reducing oxidative damage, quercetin may prevent cancer initiation and progression, particularly through mechanisms like BCL2/BAX-mediated necrosis, apoptosis, and mitotic catastrophe^102^.

One of quercetin’s potential roles in cancer prevention is via the induction of apoptosis, a programmed cell death process that is essential for eliminating cancer cells. Quercetin triggers apoptosis by activating caspase-3 and caspase-8, as well as causing PARP cleavage, which ultimately leads to the controlled demise of cancer cells^103^. This is particularly relevant for genes such as *ERBB3*, also identified in the dataset, which shows the variant rs2229046 linked to responses to trastuzumab and carboplatin in cancers like breast and ovarian cancer (Supplementary Fig. 3, Supplementary Table 7). Quercetin’s ability to modulate apoptosis and disrupt cell cycle pathways like PI3K/AKT and MAPK^104,105^ could counteract ERBB3-driven tumor proliferation.

Additionally, quercetin has been shown to cause cell cycle arrest, preventing cancer cells from proliferating. This is achieved through the modulation of key signaling pathways such as Wnt/β-catenin, PI3K/AKT, JAK/STAT, MAPK, p53, and NF-κB pathways. By disrupting these pathways, quercetin can effectively halt the uncontrolled growth and division of cancer cells. The *MTHFR* gene, also in the I4 mission, with variants like rs1801131, is involved in folate metabolism and DNA repair, both of which are critical for maintaining genomic stability under the stress of space radiation (Supplementary Fig. 3, Supplementary Table 7). Quercetin’s role in re-expressing silenced tumor suppressor genes like p16INK4a and p21 through DNA methyltransferase inhibition^106,107^ may complement the function of MTHFR and protect against cancer risks in space.

Interestingly, the *BCL2* orthologue *BAG6* has been observed to be significantly differentially expressed during Arabidopsis space flight missions^98,108^ (OSD-37, OSD-522), potentially indicating that activation of the ROS signaling pathway is common in the spaceflight environment. This observation underscores the relevance of quercetin, particularly for astronauts with genetic variants like *NOS3*, found in the I4 dataset, which regulates oxidative stress and cardiovascular health. By inhibiting enzymes such as iNOS and reducing ROS production, quercetin could further protect against the compounded oxidative stress risks associated with spaceflight.

Astronauts experience increased telomere length irrespective of mission^16,109^. One of the key characteristics of cancer cells is the upregulation of telomerase, resulting in increased telomere length, which enables tumors to bypass the Hayflick limit and continue dividing. Variants in genes like *VEGFA* and *ERBB3*, which are linked to cancer proliferation, may interact with telomerase-related pathways. Quercetin has been found to reduce telomerase activity and decrease telomere length in colon cancers, thereby suppressing malignancy^110^. Quercetin’s natural origin and lower side effects compared to conventional chemotherapy drugs make it an attractive option for cancer prevention and treatment in space, where genetic vulnerabilities and oxidative stress risks are heightened.

Lastly, quercetin also disrupts cancer cell metabolism by inhibiting the Warburg effect through downregulation of pyruvate kinase M2 (PKM2), thus impeding glycolysis and promoting oxidative phosphorylation^111–113^. This may further complement the genetic pathways influenced by variants like *VEGFA* and *ERBB3*, providing a multifaceted strategy to mitigate cancer risks in astronauts.

Several plants cultivated in space environments have shown potential as sources of quercetin. Onions, successfully grown on Salyut Space Stations from 1971 to 1981 and more recently on the Tiangong Space Station in 2023, are known for their high quercetin content (Fig. 5, Supplementary Table 2). Various types of lettuce, particularly red romaine, have been cultivated in multiple experiments aboard the International Space Station (ISS), offering another potential quercetin source. Broccoli has also been part of space agriculture experiments, including a personal project by Astronaut Don Pettit in 2012 and a probiotic-coated seed experiment on the ISS in 2018 (Supplementary Table 2). These space-grown plants, already recognized for their quercetin content on Earth, may exhibit enhanced flavonoid production in microgravity conditions, potentially providing astronauts with natural, nutrient-rich sources of this compound during extended missions.

### JAXA astronauts and mice leaky gut syndrome

**Fig. 6:**
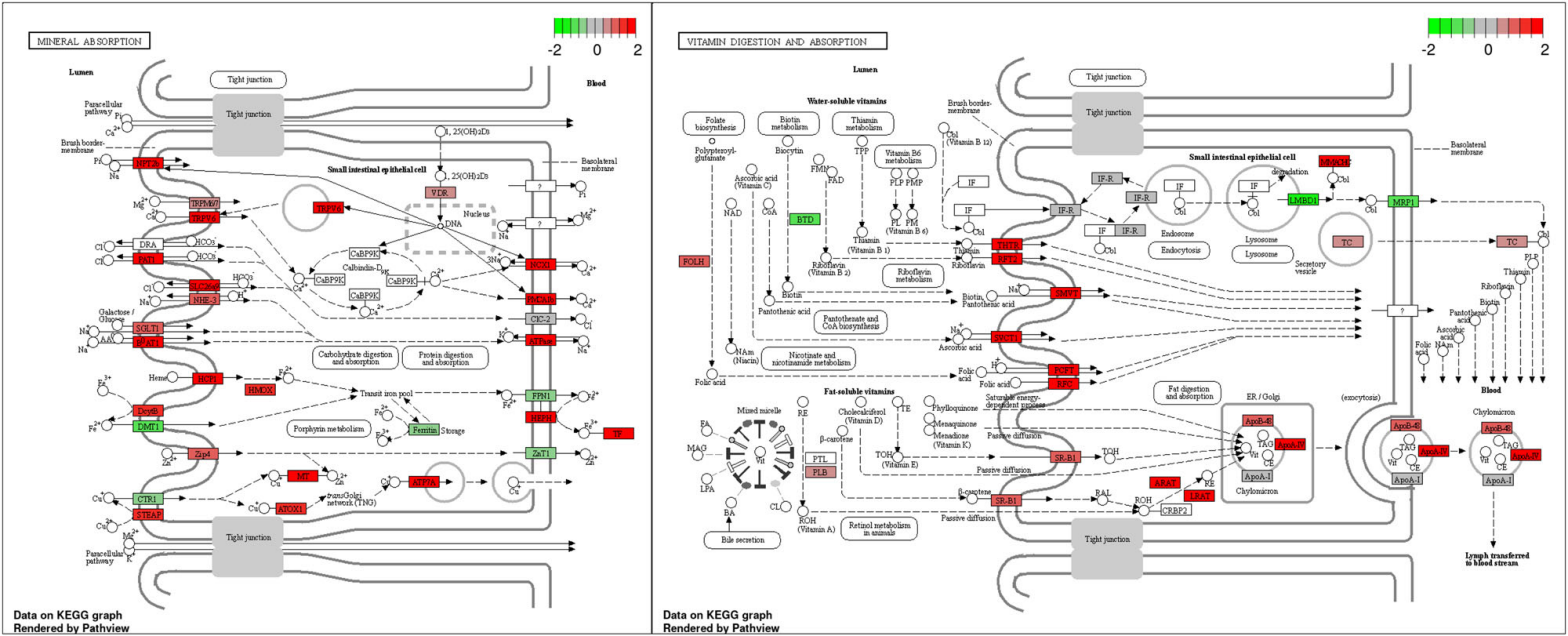
Vitamin digestion and absorption pathway dynamics on JAXA astronauts indicate leaky gut syndrome. Statistical analysis of the DESeq2 linear model (flight vs post-mission) showing pathway enrichment in the JAXA cell-free RNAseq (GLDS-530) normalized counts using the KEGG pathway database.

Cultivating suitable crops for space missions with optimal mineral and vitamin content tailored to astronaut needs is crucial. However, the effectiveness of this approach hinges on a thorough understanding of vitamin absorption in the space environment. Recent re-examination of JAXA cell-free RNA data has yielded valuable insights into the molecular processes governing intestinal permeability in microgravity (Fig. 6). The space environment presents significant challenges to human physiology, including gastrointestinal health. Recent research has highlighted the potential for spaceflight to induce leaky gut syndrome (LGS), a condition characterized by increased intestinal permeability that can have systemic effects on the body. There has historically been links between LGS and irritable bowel syndrome (IBS) and inflammatory bowel disease (IBD) in the literature^114,115^. Recently the link between LGS and IBS has been called into question though there are still proponents of the concept, however it is clear and almost universally accepted that intestinal permeability plays a large part in the pathogenesis of IBD in conjunction with immune dysfunction^116^.

The intestinal barrier is a complex structure crucial for health, impacting nutrition, immunology, microbiology, and neurological function^117^. It starts with a mucous layer that protects the intestinal wall, lubricates fecal matter, and creates a habitat for beneficial bacteria. These bacteria help prevent harmful microbes from degrading the mucous layer and maintain a nutrient sink. The epithelial layer beneath is highly structured, featuring tight junctions that block large particles while allowing smaller molecules, like water, to pass. This layer contains nutrient transporters and enzymes that minimize nutrient load, preventing bacterial overgrowth. Beyond the epithelium lies a network of immune cells, including specialized intraepithelial lymphocytes, which are phagocytic, have a high activation threshold and help preserve barrier integrity while removing larger particles that breach the mucous and epithelial layers.

Akinsuyi et al.^118^ conducted a comprehensive analysis of transcriptomic and metagenomic data from both human and murine samples to investigate the onset of leaky gut during space missions. Their study utilized JAXA cell-free RNA data and multiple RNA-Seq and microbial metagenomics datasets. The researchers identified differential expression of genes crucial for maintaining gut barrier integrity. These genes are involved in various processes, including Goblet cell differentiation, mucin production, Mucin modifications (fucosylation, glycosylation, remodeling, sialylation, and sulfation), formation of tight junction proteins, regulation of epithelial barrier integrity, Lipopolysaccharide (LPS) recognition and response and general maintenance of intestinal integrity. When this data is combined with histological and physiological data showing significant loss of immune cells in the bowel wall in space flight and bacterial penetrance into the intestinal interstitial space in simulated microgravity, points to a major potential issue for space health^119,120^. Our reanalysis of OSD-530 adds significantly to the leaky gut syndrome hypothesis in space flight and suggests that it associated with hypoxia signaling and associated with changes in vitamin and nutrient sorption function (Fig. 6).

Any countermeasure developed to address the issue of leaky gut needs to address multiple factors simultaneously. The first would be the restoration of the intestinal barrier through support/stabilization of the mucous layer, protecting epithelial cell-cell junctions, or both. The second factor is the regulation of tissue resident immune cell function to prevent aberrant responses to normal gut flora. Thankfully, there is a significant body of literature that outlines the effects of many nutritional components on gut barrier function, and many have overlaps with intestinal immune function as well.

Vitamins A and D have both been shown to increase the expression of ZO-1 in intestinal epithelial cells in vitro and vitamin D receptor deficient mice have more severe symptoms when subjected to models of intestinal inflammation^117^. The worsened inflammation in those models is linked to changes in regulatory T-cell development, thereby allowing inflammation to proceed unchecked. B vitamins, or at least deficiencies of B vitamins, has been shown to cause disruptions in intestinal epithelial barrier linked to both immune dysfunction and changes in glycolytic pathways that would affect mucus production^121^.

Switching focus to amino acids there are numerous reviews and studies, both in vitro and in vivo, that show glutamate, tryptophan, and its metabolites such as indole, and histidine support intestinal health. Glutamate and tryptophan have both been shown to preserve intestinal epithelial barrier in animals subjected to inflammatory stimuli^117,122^. Again, like vitamin D, these effects seem to be multifactorial in that these amino acids act on both the epithelial cells and the immune cells in the tissue to regulate inflammatory responses. Histidine is unique in the gastrointestinal track; it is converted to histamine and unlike other mucosal tissues in which it elicits an inflammatory response. Here it acts as a factor that maintains quiescent states in tissue resident T-cells and macrophages^123^. It also supports intestinal lymphatic pumping function which helps remove any potential inflammatory substances from the tissue along with fat soluble nutrients^124^.

Lastly, there is role of short chain fatty acids (SCFAs) which are predominantly produced by the microbiome when appropriate substrates are provided. Chief among these is butyrate, which has been shown in numerous models of intestinal inflammation to downregulate inflammatory pathways in tissue resident immune cells and help maintain a quiescent state under normal conditions^125^. In addition to this there are the links between SCFAs and the gut brain axis where shifts in proportions of certain SCFAs can predispose an individual to anxiety which is in turn linked to intestinal inflammation and permeability^126^.

With the wide variety and impacts of nutritional components on intestinal health it seems a likely area to exploit as a preventative or counter measure to gastrointestinal issues during exploration and colonization class missions. Identifying food stuffs that are rich in the aforementioned nutrients, can be readily grown, and resistant to the challenges of the space and extraterrestrial environment should be a primary goal for agencies looking towards long duration, self-sustaining missions.

## Methods

### Data analysis

Data was gathered from the Space Omics and Medical Atlas (SOMA) database, incorporating datasets from the National Aeronautics and Space Administration (NASA) Twins Study, the Japan Aerospace Exploration Agency (JAXA) Cell-Free Epigenome (CFE), and SpaceX Inspiration4. The data was downloaded and organized according to the following variables: Gene, *p*-value, *q*-value, Log2foldchange and Cell type.

KEGG pathway enrichment was completed with ShinyGO 0.80^127^.

DEGs were clustered according to broader contrasts (“Post vs Pre”, “Post vs. Ground”, “Flight” (for all in-flight comparisons), “Flight vs Ground”, and “Flight vs Post”) and broader cell type groups (“B Cell”, “Dendritic Cell”, “LD”, “Monocyte”, “Natural Killer Cell”, “PBMC”, “T Cell”, and “Other”) with mean log2FC calculated for duplicate genes within a cluster^128,129^.

Sankey diagrams were generated to visualize relationships between studies, cell types, and contrasts, with one diagram incorporating genes and log2FC values. Sankey diagrams were generated to visualize relationships between studies, cell types, and contrasts, with one diagram incorporating genes and log2FC values. Input data were processed using Python (version 3.9.21), with pandas (version 2.2.3) for data manipulation, NumPy (version 2.0.2) for numerical operations, and Matplotlib (version 3.9.4) for colormap normalization. Plotly (version 6.0.0) was used as the primary visualization library to construct interactive Sankey diagrams, with uniform weights assigned to each link and log2FC values mapped to color gradients. Figures were exported using Kaleido (version 0.2.1).

### Mitochondrial pathways

The genes were selected from the MitoCarta3.0, a resource from the Broad Institute, which provides a list of genes that strongly indicate mitochondrial localization. The MitoPathways3.0 file contains the genes that have been manually curated into 149 mitochondrial pathways. Here only those metabolic pathways in which vitamins or micronutrients are involved were selected. The desired genes were filtered according to their involvement in the vitamin and micronutrient metabolism. For each vitamin and micronutrient, the corresponding genes from the MitoPathways3.0 list were entered individually into the Human Gene Expression section of the SOMA database. The used datasets were from the National Aeronautics and Space Administration (NASA) Twins Study, the Japan Aerospace Exploration Agency (JAXA) Cell-Free Epigenome (CFE), and SpaceX Inspiration4 Multiome RNA data. To ensure statistical significance only results with following parameters were selected: Log2foldchange > +/− 1 and *p*-and *q*-values under 0.05. Furthermore, only Pre- vs. Post-Flight data were included.

### Vitamin absorption pathway analysis

Pathway analyses were performed using fold-change values returned by limma or DESeq2 for differential expression analysis^130^. Since fold-change values were obtained for each linear model comparison, pathway analysis was conducted separately for each comparison. It is important to note that pathway analysis utilizes fold-change values for all genes, making it independent of the selected differentially expressed genes (DEGs).

### Nutrition and pharmacogenomic associated variants from Inspiration4 crew

Whole genome sequencing was performed before and after flight during the Inspiration4 mission as described in ref. ^17^. We performed data pre-processing with GATK4 Best Practice, followed by variant calling on the crew genomes using two independent variant callers, Strelka2 and DeepVariant. Genome annotation was performed by Ensembl VEP and consensus variants between variant callers for each astronaut were compiled into a single list.

To find specific variants of interest in the crew, we first identified the most commonly prescribed medicines in U.S. and European emergency departments and utilized PharmGKB to generate a list of notable variants associated with these drugs. The notable drug-associated variants were then mapped back to the Inspiration4 crew genomes in an anonymized report.

### Figure generation

Some figures were generated partially using BioRender. Statistical plots were produced with GraphPad Prism 10, and network visualizations were created using Cytoscape v3.10.3.

## Supplementary information

Supplementary material

Supplementary table 4

## Data Availability

The study utilized multiple Open Science Data Repository (OSR) databases to compile comprehensive research across plant and human spaceflight studies. These databases include OSD-269 for lettuce nutritional analysis on the ISS, OSD-796 for Arabidopsis thaliana secondary metabolite research, and several OSD datasets (OSD-569, OSD-570, OSD-571-575) for human health investigations from missions like Inspiration4 and the NASA Twins Study. The diverse datasets encompass nutritional, molecular, physiological, and multi-omics profiles from both plant and human spaceflight experiments, providing a holistic view of biological responses to space environments. Data was gathered from the Space Omics and Medical Atlas (SOMA) database, incorporating datasets from the National Aeronautics and Space Administration (NASA) Twins Study, the Japan Aerospace Exploration Agency (JAXA) Cell-Free Epigenome (CFE), and SpaceX Inspiration4. The data was downloaded and organized according to the following variables: Gene, *p*-value, *q*-value, Log2foldchange and Cell type.

## References

[1] Pittia, P. & Heer, M. Space food for the future: nutritional challenges and technological strategies for healthy and high-quality products. In *Space Manufacturing and Resources: Earth and Planetary Exploration Applications* 251–268 10.1002/9783527830909.CH13 (2022).

[2] Wheeler, R. M. Horticulture for mars. *Acta Hortic.* **642**, 201–215 (2004).

[3] Monje, O., Stutte, G. W., Goins, G. D., Porterfield, D. M. & Bingham, G. E. Farming in space: Environmental and biophysical concerns. *Adv. Space Res.* **31**, 151–167 (2003). 12577999 10.1016/s0273-1177(02)00751-2

[4] Droppert, P. M. The effects of microgravity on the skeletal system-a review. *J. Br. Interplanet. Soc.* **43**, 19–24 (1990). 12856692

[5] Paulsen, K. et al. Microgravity-induced alterations in signal transduction in cells of the immune system. *Acta Astronaut* **67**, 1116–1125 (2010).

[6] Antonutto, G. & di Prampero, P. E. Cardiovascular deconditioning in microgravity: some possible countermeasures. *Eur. J. Appl. Physiol.* **90**, 283–291 (2003). 12851824 10.1007/s00421-003-0884-5

[7] Jha, R. et al. Global nutritional challenges and opportunities: Buckwheat, a potential bridge between nutrient deficiency and food security. *Trends Food Sci. Technol.* **145**, 104365 (2024).

[8] Cooper, M., Perchonok, M. & Douglas, G. L. Initial assessment of the nutritional quality of the space food system over three years of ambient storage. *npj Microgravity* **3**, 1–4 (2017). 28649639 10.1038/s41526-017-0022-z PMC5466603

[9] Wei, L. J. et al. Analysis of cytogenetic damage in rice seeds induced by energetic heavy ions on-ground and after spaceflight. *J. Radiat. Res.* **47**, 273–278 (2006). 16974070 10.1269/jrr.0613

[10] De Micco, V., Arena, C., Pignalosa, D. & Durante, M. Effects of sparsely and densely ionizing radiation on plants. *Radiat. Environ. Biophys.* **50**, 1–19 (2011). 21113610 10.1007/s00411-010-0343-8

[11] Kranz, A. R. Genetic and physiological damage induced by cosmic radiation on dry plant seeds during space flight. *Adv. Space Res.* **6**, 135–138 (1986). 11537811 10.1016/0273-1177(86)90076-1

[12] Dedolph, R. R. The influence of simulated low-gravity environments on growth, development and metabolism of plants. *Life Sci. Space Res.* **5**, 217–228 (1967). 11973847

[13] Wolff, S. A., Coelho, L. H., Zabrodina, M., Brinckmann, E. & Kittang, A. I. Plant mineral nutrition, gas exchange and photosynthesis in space: a review. *Adv. Space Res.* **51**, 465–475 (2013).

[14] Carillo, P., Morrone, B., Fusco, G. M., De Pascale, S. & Rouphael, Y. Challenges for a sustainable food production system on board of the International Space Station: a technical review. *Agronomy* **10**, 687 (2020).

[15] Khodadad, C. L. M. et al. Microbiological and nutritional analysis of lettuce crops grown on the International Space Station. *Front Plant Sci.* **11**, 505516 (2020). 10.3389/fpls.2020.00199 PMC7067979 32210992

[16] Garrett-Bakelman, F. E. et al. The NASA twins study: a multidimensional analysis of a year-long human spaceflight. *Science* **364**, 6436 (2019). 10.1126/science.aau8650 PMC7580864 30975860

[17] Jones, C. W. et al. Molecular and physiological changes in the SpaceX Inspiration4 civilian crew. *Nature* **632**, 1155–1164 (2024). 38862026 10.1038/s41586-024-07648-x PMC11357997

[18] Overbey, E. G. et al. The Space Omics and Medical Atlas (SOMA) and International Astronaut Biobank. *Nature* **632**, 1145–1154 (2024). 38862028 10.1038/s41586-024-07639-y PMC11357981

[19] Larina, I. M. & Verigo, V. V. Calcium metabolism and a mars mission: new problems. *Hum. Physiol.* **29**, 470–475 (2003). 13677204

[20] Belyavskaya, N. A. Free and membrane-bound calcium in microgravity and microgravity effects at the membrane level. *Adv. Space Res.* **17**, 169–177 (1996). 11538612 10.1016/0273-1177(95)00631-n

[21] Kordyum, E. L. & Chapman, D. K. Plants and microgravity: patterns of microgravity effects at the cellular and molecular levels. *Cytol. Genet.* **51**, 108–116 (2017). 30484618

[22] Bangerth, F. Calcium-related physiological disorders of plants. *Annu. Rev. Phytopathol.* **17**, 97–122 (1979).

[23] Nordin, B. E. C. & Morris, H. A. The calcium deficiency model for osteoporosis. *Nutr. Rev.* **47**, 65–72 (1989). 2649803 10.1111/j.1753-4887.1989.tb02794.x

[24] Meyers, A. & Wyatt, S. E. Plant space biology in the genomics. *Age. Annu. Plant Rev. Online* **5**, 123–150 (2022).

[25] Shen, Y. et al. Research on lettuce growth technology onboard Chinese Tiangong II Spacelab. *Acta Astronaut* **144**, 97–102 (2018).

[26] Massa, G. D. et al. VEG-01: Veggie hardware validation testing on the International Space Station. *Open Agric.* **2**, 33–41 (2017).

[27] Stutte, G. W., Newsham, G., Morrow, R. M. & Wheeler, R. M. Operational evaluation of VEGGIE food production system in the Habitat Demonstration Unit. In *41st International Conference on Environmental Systems 2011, ICES 2011*10.2514/6.2011-5262 (2011)

[28] Udensi, U. K. & Tchounwou, P. B. Potassium homeostasis, oxidative stress, and human disease. *Int. J. Clin. Exp. Physiol.* **4**, 111 (2017). 29218312 10.4103/ijcep.ijcep_43_17 PMC5716641

[29] Biver, E. et al. Dietary recommendations in the prevention and treatment of osteoporosis. *Joint Bone Spine* **90**, 105521 (2023). 10.1016/j.jbspin.2022.105521 36566976

[30] Blaszczyk, U. & Duda-Chodak, A. Magnesium: its role in nutrition and carcinogenesis. *Rocz. Panstw. Zakl. Hig.* **64**, 165–171 (2013). 24325082

[31] Zwart, S. R., Morgan, J. L. L. & Smith, S. M. Iron status and its relations with oxidative damage and bone loss during long-duration space flight on the International Space Station. *Am. J. Clin. Nutr.* **98**, 217–223 (2013). 23719548 10.3945/ajcn.112.056465

[32] Chausow, D. G. & Czarnecki-Maulden, G. L. The relative bioavailability of plant and animal sources of iron to the cat and chick. *Nutr. Res.* **8**, 1041–1050 (1988).

[33] Shipp, J. & Abdel-Aal, E.-S. M. Food applications and physiological effects of anthocyanins as functional food ingredients. *AACE Clin. Case Rep.* **7**, 1 (2021).33718602

[34] Yan, Y. & Li, J. Association of dietary anthocyanidins intake with all-cause mortality and cardiovascular diseases mortality in USA adults: a prospective cohort study. *Sci. Rep.* **14**, 1–12 (2024). 39496659 10.1038/s41598-024-76805-z PMC11535342

[35] Williamson, G. The role of polyphenols in modern nutrition. *Nutr. Bull.* **42**, 226–235 (2017). 28983192 10.1111/nbu.12278 PMC5601283

[36] Kyoung Chun, O. et al. Daily consumption of phenolics and total antioxidant capacity from fruit and vegetables in the American diet. *J. Sci. Food Agric.* **85**, 1715–1724 (2005).

[37] Chibisov, S. et al. Polyphenolics and flavonoids in health and diseases. In *Functional Foods and Nutraceuticals in Metabolic and Non-communicable Diseases* 671–689 10.1016/B978-0-12-819815-5.00016-1 (2022).

[38] Rodriguez, A. et al. Raman spectroscopy as a tool for assessing plant growth in space and on lunar regolith simulants. *npj Microgravity* **11**, 19 (2025). 40425596 10.1038/s41526-025-00479-8 PMC12117163

[39] Barcenilla, B. B. et al. Arabidopsis telomerase takes off by uncoupling enzyme activity from telomere length maintenance in space. *Nat. Commun.* **14**, 7854 (2023). 38030615 10.1038/s41467-023-41510-4 PMC10686995

[40] Kohli, S. K. et al. ROS signaling in plants under heavy metal stress. In *Reactive Oxygen Species and Antioxidant Systems in Plants: Role and Regulation under Abiotic Stress* 185–214 10.1007/978-981-10-5254-5_8 (2017).

[41] Sharma, A. et al. Pesticides-mediated ROS generation in plants. *Pesticides in the Environment* 179–202 10.1016/B978-0-323-99427-9.00001-X (2024).

[42] Stein, T. P. Space flight and oxidative stress. *Nutrition* **18**, 867–871 (2002). 12361781 10.1016/s0899-9007(02)00938-3

[43] Goodenow-Messman, D. A., Gokoglu, S. A., Kassemi, M. & Myers, J. G. Numerical characterization of astronaut CaOx renal stone incidence rates to quantify in-flight and post-flight relative risk. *npj Microgravity 2022 8:1* **8**, 1–17 (2022). 10.1038/s41526-021-00187-z PMC8799707 35091560

[44] Whitson, P. A., Pietrzyk, R. A. & Pak, C. Y. C. Renal stone risk assessment during space shuttle flights. *J. Urol.* **158**, 2305–2310 (1997). 9366381 10.1016/s0022-5347(01)68240-5

[45] Holick, M. F. Sunlight and vitamin D for bone health and prevention of autoimmune diseases, cancers, and cardiovascular disease. *Am. J. Clin. Nutr.* **80**, 1678S–1688S (2004). 15585788 10.1093/ajcn/80.6.1678S

[46] Reichrath, J. The challenge resulting from positive and negative effects of sunlight: How much solar UV exposure is appropriate to balance between risks of vitamin D deficiency and skin cancer?. *Prog. Biophys. Mol. Biol.* **92**, 9–16 (2006). 16603232 10.1016/j.pbiomolbio.2006.02.010

[47] Blumenthal, R. D. et al. Anti-oxidant vitamins reduce normal tissue toxicity induced by radio-immunotherapy. *Int. J. Cancer* **86**, 276–280 (2000). 10738257 10.1002/(sici)1097-0215(20000415)86:2<276::aid-ijc19>3.0.co;2-5

[48] Bhuiyan, A. A., Dash, S. K., Shahriar, S. M. H., Nahid, F. & Arefin, S. A case of sub-acute combined degeneration of the spinal cord with associated pernicious anaemia. *Pulse* **5**, 57–60 (2011).

[49] Smith, S. M. et al. Space flight calcium: implications for astronaut health, spacecraft operations, and earth. *Nutrients* **4**, 2047–2068 (2012). 23250146 10.3390/nu4122047 PMC3546622

[50] Zittermann, A. et al. Microgravity inhibits intestinal calcium absorption as shown by a stable strontium test. *Eur. J. Clin. Invest.* **30**, 1036–1043 (2000). 11122318 10.1046/j.1365-2362.2000.00682.x

[51] Gundberg, C. M., Looker, A. C., Nieman, S. D. & Calvo, M. S. Patterns of osteocalcin and bone specific alkaline phosphatase by age, gender, and race or ethnicity. *Bone* **31**, 703–708 (2002). 12531565 10.1016/s8756-3282(02)00902-x

[52] Henry, Y. M. & Eastell, R. Ethnic and gender differences in bone mineral density and bone turnover in young adults: Effect of bone size. *Osteoporos. Int.* **11**, 512–517 (2000). 10982167 10.1007/s001980070094

[53] Siew, K. et al. Cosmic kidney disease: an integrated pan-omic, physiological and morphological study into spaceflight-induced renal dysfunction. *Nat. Commun.* **15**, 1–20 (2024). 38862484 10.1038/s41467-024-49212-1 PMC11167060

[54] Wang, S. et al. An insertion/deletion polymorphism within 3′UTR of RYR2 modulates sudden unexplained death risk in Chinese populations. *Forensic. Sci. Int.* **270**, 165–172 (2017). 27987400 10.1016/j.forsciint.2016.12.005

[55] Wysocki, C. et al. A human ITPR3 variant causes a dominant negative attenuation of calcium responses with immunodeficiency and growth delay confirmed in a mouse model. *Clin. Immunol.* **250**, 109379 (2023).

[56] Nemoto, E. et al. Wnt5a signaling is a substantial constituent in bone morphogenetic protein-2-mediated osteoblastogenesis. *Biochem. Biophys. Res. Commun.* **422**, 627–632 (2012). 22609204 10.1016/j.bbrc.2012.05.039

[57] Roberts, J. L. et al. Deletion of Wnt5a in osteoclasts results in bone loss through decreased bone formation. *Ann. N. Y Acad. Sci.* **1463**, 45–59 (2020). 31919867 10.1111/nyas.14293

[58] Erben, R. G. Physiological actions of fibroblast growth factor-23. *Front. Endocrinol.* **9**, 267 (2018). 10.3389/fendo.2018.00267 PMC5985418 29892265

[59] Park, E. J., Truong, V. L., Jeong, W. S. & Min, W. K. Brain-derived neurotrophic factor (BDNF) enhances osteogenesis and may improve bone microarchitecture in an ovariectomized rat model. *Cells* **13**, 518 (2024). 38534361 10.3390/cells13060518 PMC10969057

[60] Parker, R. S. et al. Role of the neurologic system in fracture healing: an extensive review. *Curr. Osteoporos. Rep.* **22**, 205–216 (2024). 38236509 10.1007/s11914-023-00844-0 PMC10912173

[61] Brakspear, K. S. & Mason, D. J. Glutamate signaling in bone. *Front Endocrinol.* **3**, 28971 (2012). 10.3389/fendo.2012.00097 PMC3412269 22888325

[62] Yoon, S. H., Ryu, J. Y., Lee, Y., Lee, Z. H. & Kim, H. H. Adenylate cyclase and calmodulin-dependent kinase have opposite effects on osteoclastogenesis by regulating the PKA-NFATc1 pathway. *J. Bone Miner. Res.* **26**, 1217–1229 (2011). 21611964 10.1002/jbmr.310

[63] Zhang, Y., Yang, J., Wang, X. & Li, X. GNG7 and ADCY1 as diagnostic and prognostic biomarkers for pancreatic adenocarcinoma through bioinformatic-based analyses. *Sci. Rep.* **11**, 1–13 (2021). 34650124 10.1038/s41598-021-99544-x PMC8516928

[64] Simic, P. & Babitt, J. L. Regulation of FGF23: beyond bone. *Curr. Osteoporos. Rep.* **19**, 563–573 (2021). 34757587 10.1007/s11914-021-00703-w PMC8958553

[65] Mao, D., Epple, H., Uthgenannt, B., Novack, D. V. & Faccio, R. PLCγ2 regulates osteoclastogenesis via its interaction with ITAM proteins and GAB2. *J. Clin. Invest* **116**, 2869–2879 (2006). 17053833 10.1172/JCI28775 PMC1616195

[66] Kobayashi, Y., Uehara, S., Udagawa, N. & Takahashi, N. Regulation of bone metabolism by Wnt signals. *J. Biochem.* **159**, 387–392 (2016). 26711238 10.1093/jb/mvv124 PMC4885935

[67] Weitzmann, M. N. & Pacifici, R. The role of T lymphocytes in bone metabolism. *Immunol. Rev.* **208**, 154–168 (2005). 16313347 10.1111/j.0105-2896.2005.00324.x

[68] Yasuda, S. et al. Dystrophic heart failure blocked by membrane sealant poloxamer. *Nature* **436**, 1025–1029 (2005). 16025101 10.1038/nature03844

[69] Teitelbaum, S. L. Bone resorption by osteoclasts. *Science* **289**, 1504–1508 (2000). 10968780 10.1126/science.289.5484.1504

[70] Devasani, K. & Yao, Y. Expression and functions of adenylyl cyclases in the CNS. *Fluids Barriers CNS* **19**, 1–18 (2022). 35307032 10.1186/s12987-022-00322-2 PMC8935726

[71] Szymanowicz, O. et al. A review of the CACNA gene family: its role in neurological disorders. *Diseases* **12**, 90 (2024). 38785745 10.3390/diseases12050090 PMC11119137

[72] Thapa, S. & Cao, X. Nervous regulation: beta-2-adrenergic signaling in immune homeostasis, cancer immunotherapy, and autoimmune diseases. *Cancer Immunol. Immunother.* **72**, 2549–2556 (2023). 37060364 10.1007/s00262-023-03445-z PMC10693916

[73] Stallaert, W. et al. Purinergic receptor transactivation by the β2-adrenergic receptor increases intracellular Ca2+ in nonexcitable cells. *Mol. Pharm.* **91**, 533–544 (2017). 10.1124/mol.116.106419 28280061

[74] Kongsbak, M., Levring, T. B., Geisler, C. & von Essen, M. R. The vitamin D receptor and T cell function. *Front. Immunol.* **4**, 51253 (2013). 10.3389/fimmu.2013.00148 PMC3684798 23785369

[75] Wright, C. S., Robling, A. G., Farach-Carson, M. C. & Thompson, W. R. Skeletal functions of voltage sensitive calcium channels. *Curr. Osteoporos. Rep.* **19**, 206–221 (2021). 33721180 10.1007/s11914-020-00647-7 PMC8216424

[76] Pitt, G. S., Matsui, M. & Cao, C. Voltage-gated calcium channels in nonexcitable tissues. *Annu. Rev. Physiol.* **83**, 183–203 (2021). 33106102 10.1146/annurev-physiol-031620-091043 PMC8281591

[77] Smith, S. M. et al. Calcium metabolism before, during, and after a 3-mo spaceflight: Kinetic and biochemical changes. *Am. J. Physiol. Regul. Integr. Comp. Physiol.* **277**, R1–10 (1999). 10.1152/ajpregu.1999.277.1.r1 10409251

[78] Mair, D. B. et al. Spaceflight-induced contractile and mitochondrial dysfunction in an automated heart-on-a-chip platform. *Proc. Natl Acad. Sci. USA* **121**, e2404644121 (2024). 10.1073/pnas.2404644121 PMC11459163 39312653

[79] Rudolf, A. M. & Hood, W. R. Mitochondrial stress in the spaceflight environment. *Mitochondrion* **76**, 101855 (2024). 10.1016/j.mito.2024.101855 38403094

[80] da Silveira, W. A. et al. Comprehensive multi-omics analysis reveals mitochondrial stress as a central biological hub for spaceflight impact. *Cell* **183**, 1185–1201.e20 (2020). 33242417 10.1016/j.cell.2020.11.002 PMC7870178

[81] Davey, G. P. & Bolaños, J. P. Peroxiredoxin 5 links mitochondrial redox signalling with calcium dynamics: impact on Parkinson’s disease. *J. Neurochem.* **125**, 332–333 (2013). 23442153 10.1111/jnc.12171

[82] Maio, N., Jain, A. & Rouault, T. A. Mammalian iron–sulfur cluster biogenesis: Recent insights into the roles of frataxin, acyl carrier protein and ATPase-mediated transfer to recipient proteins. *Curr. Opin. Chem. Biol.* **55**, 34–44 (2020). 31918395 10.1016/j.cbpa.2019.11.014 PMC7237328

[83] Stroud, D. A. et al. Accessory subunits are integral for assembly and function of human mitochondrial complex I. *Nature* **538**, 123–126 (2016). 27626371 10.1038/nature19754

[84] Hou, T. et al. NDUFAB1 confers cardio-protection by enhancing mitochondrial bioenergetics through coordination of respiratory complex and supercomplex assembly. *Cell Res.* **29**, 754–766 (2019). 31366990 10.1038/s41422-019-0208-x PMC6796901

[85] Zhang, R., Hou, T., Cheng, H. & Wang, X. NDUFAB1 protects against obesity and insulin resistance by enhancing mitochondrial metabolism. *FASEB J.* **33**, 13310–13322 (2019). 31530015 10.1096/fj.201901117RR PMC6894049

[86] Kim, M. J., Moon, Y., Tou, J. C., Mou, B. & Waterland, N. L. Nutritional value, bioactive compounds and health benefits of lettuce (Lactuca sativa L.). *J. Food Composition Anal.* **49**, 19–34 (2016).

[87] Cohu, C. M., Lombardi, E., Adams, W. W. & Demmig-Adams, B. Increased nutritional quality of plants for long-duration spaceflight missions through choice of plant variety and manipulation of growth conditions. *Acta Astronaut* **94**, 799–806 (2014).

[88] Chunxiao, X. & Hong, L. Crop candidates for the bioregenerative life support systems in China. *Acta Astronaut* **63**, 1076–1080 (2008).

[89] Houschyar, K. S. et al. Wnt pathway in bone repair and regeneration - what do we know so far. *Front. Cell Dev. Biol.* **6**, 170 (2019). 10.3389/fcell.2018.00170 PMC6330281 30666305

[90] Wang, B. et al. Quercetin regulates calcium and phosphorus metabolism through the Wnt signaling pathway in broilers. *Front. Vet. Sci.* **8**, 786519 (2022). 35155643 10.3389/fvets.2021.786519 PMC8828646

[91] Yamaguchi, M. & Weitzmann, M. N. Vitamin K2 stimulates osteoblastogenesis and suppresses osteoclastogenesis by suppressing NF-κB activation. *Int. J. Mol. Med.* **27**, 3–14 (2011). 21072493 10.3892/ijmm.2010.562

[92] Deng, T. T. et al. Pharmacological and mechanistic aspects of quercetin in osteoporosis. *Front. Pharm.* **15**, 1338951 (2024). 10.3389/fphar.2024.1338951 PMC10851760 38333006

[93] Hu, G. et al. Glutaminolysis provides nucleotides and amino acids to regulate osteoclast differentiation in mice. *EMBO Rep.* **25**, 4515–4541 (2024). 10.1038/s44319-024-00255-x PMC11467445 39271775

[94] Burgess, A. J., Pranggono, R., Escribà-Gelonch, M. & Hessel, V. Biofortification for space farming: Maximising nutrients using lettuce as a model plant. *Fut. Foods* **9**, 100317 (2024).

[95] Xiong, Y., Hirano, H., Lane, N. E., Nandi, S. & McDonald, K. A. Plant-based production and characterization of a promising Fc-fusion protein against microgravity-induced bone density loss. *Front. Bioeng. Biotechnol.* **10**, 962292 (2022). 36172011 10.3389/fbioe.2022.962292 PMC9511166

[96] Barker, R. et al. Meta-analysis of the space flight and microgravity response of the Arabidopsis plant transcriptome. *NPJ Microgravity* **9**, 21 (2023). 10.1038/s41526-023-00247-6 PMC10027818 36941263

[97] Kruse, C. P. S. et al. Spaceflight induces novel regulatory responses in Arabidopsis seedling as revealed by combined proteomic and transcriptomic analyses. *BMC Plant. Biol.* **20**, 237 (2020). 10.1186/s12870-020-02392-6 PMC7251690 32460700

[98] Choi, W.-G. et al. Variation in the transcriptome of different ecotypes of Arabidopsis thaliana reveals signatures of oxidative stress in plant responses to spaceflight. *Am. J. Bot.* **106**, 123–136 (2019). 30644539 10.1002/ajb2.1223

[99] Baqer, S. H., Al-Shawi, S. G. & Al-Younis, Z. K. Quercetin, the potential powerful flavonoid for human and food: a review. *Front. Biosci.* **16**, 30 (2024). 10.31083/j.fbe1603030 39344383

[100] Zhang, L. et al. New perspectives on the therapeutic potential of quercetin in non-communicable diseases: Targeting Nrf2 to counteract oxidative stress and inflammation. *J. Pharm. Anal.* **14**, 100930 (2024). 39005843 10.1016/j.jpha.2023.12.020 PMC11245930

[101] Chaudhary, S., Sharma, S., Fuloria, S. & Sharma, P. K. Anti-inflammatory and anti-arthritis activity of quercetin: a comprehensive review. *Curr. Rheumatol. Rev.* **21**, 144–159 (2024). 10.2174/0115733971280645240415101912 38808723

[102] Klimaszewska-Wiśniewska, A. et al. Antiproliferative and antimetastatic action of quercetin on A549 non-small cell lung cancer cells through its effect on the cytoskeleton. *Acta Histochem.* **119**, 99–112 (2017). 27887793 10.1016/j.acthis.2016.11.003

[103] Baruah, M. M., Khandwekar, A. P. & Sharma, N. Quercetin modulates Wnt signaling components in prostate cancer cell line by inhibiting cell viability, migration, and metastases. *Tumor Biol.* **37**, 14025–14034 (2016). 10.1007/s13277-016-5277-6 27495232

[104] Seo, H. S. et al. Quercetin induces caspase-dependent extrinsic apoptosis through inhibition of signal transducer and activator of transcription 3 signaling in HER2-overexpressing BT-474 breast cancer cells. *Oncol. Rep.* **36**, 31–42 (2016). 27175602 10.3892/or.2016.4786 PMC4899028

[105] Caddeo, C. et al. Effect of quercetin and resveratrol co-incorporated in liposomes against inflammatory/oxidative response associated with skin cancer. *Int. J. Pharm.* **513**, 153–163 (2016). 27609664 10.1016/j.ijpharm.2016.09.014

[106] Jiang, W. et al. Remodeling the epigenetic landscape of cancer—application potential of flavonoids in the prevention and treatment of cancer. *Front. Oncol.* **11**, 705903 (2021). 34235089 10.3389/fonc.2021.705903 PMC8255972

[107] Bouyahya, A. et al. The role of epigenetic modifications in human cancers and the use of natural compounds as epidrugs: mechanistic pathways and pharmacodynamic actions. *Biomolecules* **12**, 367 (2022). 35327559 10.3390/biom12030367 PMC8945214

[108] Olanrewaju, G. O. et al. Integrative transcriptomics and proteomics profiling of Arabidopsis thaliana elucidates novel mechanisms underlying spaceflight adaptation. *Front. Plant Sci.* **14**, 1260429 (2023). 38089794 10.3389/fpls.2023.1260429 PMC10712242

[109] Luxton, J. J. et al. Temporal telomere and DNA damage responses in the space radiation environment. *Cell Rep.* 108435 10.1016/j.celrep.2020.108435 (2020). 10.1016/j.celrep.2020.108435 33242411

[110] Bhatiya, M., Pathak, S., Jothimani, G., Duttaroy, A. K. & Banerjee, A. A comprehensive study on the anti-cancer effects of quercetin and its epigenetic modifications in arresting progression of colon cancer cell proliferation. *Arch. Immunol. Ther. Exp.* **71**, 6 (2023). 10.1007/s00005-023-00669-w PMC9941246 36807774

[111] El-Far, A. H., Al Jaouni, S. K., Li, X. & Fu, J. Cancer metabolism control by natural products: Pyruvate kinase M2 targeting therapeutics. *Phytother. Res.* **36**, 3181–3201 (2022). 35794729 10.1002/ptr.7534

[112] Monteiro, F., Shetty, S. S. & Kumari N, S. Natural antioxidants as inhibitors of pyruvate kinase M2 in Warburg phenotypes. *J. Herb. Med.* **42**, 100750 (2023).

[113] Reyes-Farias, M. & Carrasco-Pozo, C. The anti-cancer effect of quercetin: molecular implications in cancer metabolism. *Int. J. Mol. Sci.* **20**, 3177 (2019). 31261749 10.3390/ijms20133177 PMC6651418

[114] Inczefi, O., Bacsur, P., Resál, T., Keresztes, C. & Molnár, T. The influence of nutrition on intestinal permeability and the microbiome in health and disease. *Front. Nutr.* **9**, 718710 (2022). 35548572 10.3389/fnut.2022.718710 PMC9082752

[115] Awad, K. et al. Epithelial barrier dysfunction in diarrhea-predominant irritable bowel syndrome (IBS-D) via downregulation of claudin-1. *Cells* **12**, 2846 (2023). 10.3390/cells12242846 PMC10741936 38132165

[116] Lacy, B. E., Wise, J. L. & Cangemi, D. J. Leaky gut syndrome: myths and management. *Gastroenterol. Hepatol.* **20**, 264 (2024). PMC11345991 39193076

[117] Farré, R., Fiorani, M., Rahiman, S. A. & Matteoli, G. Intestinal permeability, inflammation and the role of nutrients. *Nutrients* **12**, 1185 (2020). 32340206 10.3390/nu12041185 PMC7231157

[118] Akinsuyi, O. S., Xhumari, J., Ojeda, A. & Roesch, L. F. W. Gut permeability among Astronauts during Space missions. *Life Sci. Space Res.* **41**, 171–180 (2024). 10.1016/j.lssr.2024.03.003 38670644

[119] Cromer, W. E. & Zawieja, D. C. Acute exposure to space flight results in evidence of reduced lymph Transport, tissue fluid Shifts, and immune alterations in the rat gastrointestinal system. *Life Sci. Space Res.* **17**, 74–82 (2018). 10.1016/j.lssr.2018.03.005 29753416

[120] Zhou, Y. et al. Effect of solar particle event radiation and hindlimb suspension on gastrointestinal tract bacterial translocation and immune activation. *PLoS One* **7**, e44329 (2012). 23028522 10.1371/journal.pone.0044329 PMC3446907

[121] Hossain, K. S., Amarasena, S. & Mayengbam, S. B vitamins and their roles in gut health. *Microorganisms* **10**, 1168 (2022). 35744686 10.3390/microorganisms10061168 PMC9227236

[122] Moran, E. T. Nutrients central to maintaining intestinal absorptive efficiency and barrier integrity with fowl. *Poult. Sci.* **96**, 1348–1363 (2017). 27665014 10.3382/ps/pew337

[123] Hemarajata, P. et al. Lactobacillus reuteri-specific immunoregulatory gene rsiR modulates histamine production and immunomodulation by Lactobacillus reuteri. *J. Bacteriol.* **195**, 5567–5576 (2013). 24123819 10.1128/JB.00261-13 PMC3889603

[124] Nizamutdinova, I. T. et al. Mast cells and histamine are triggering the NF-κB-mediated reactions of adult and aged perilymphatic mesenteric tissues to acute inflammation. *Aging* **8**, 3065–3090 (2016). 27875806 10.18632/aging.101113 PMC5191886

[125] Hodgkinson, K. et al. Butyrate’s role in human health and the current progress towards its clinical application to treat gastrointestinal disease. *Clin. Nutr.* **42**, 61–75 (2023). 36502573 10.1016/j.clnu.2022.10.024

[126] Cromer, W. E., Endsley, M. & Cromer, W. E. The effects of transplantation of the spaceflight intestinal microbiome on short chain fatty acid production, lymphoid organ immune composition and transport kinetics. Preprint at 10.20944/PREPRINTS202307.1699.V1 (2023).

[127] Ge, S. X., Jung, D., Jung, D. & Yao, R. ShinyGO: a graphical gene-set enrichment tool for animals and plants. *Bioinformatics* **36**, 2628–2629 (2020). 31882993 10.1093/bioinformatics/btz931 PMC7178415

[128] Luo, W. & Brouwer, C. Pathview: an R/Bioconductor package for pathway-based data integration and visualization. *Bioinformatics* **29**, 1830–1831 (2013). 23740750 10.1093/bioinformatics/btt285 PMC3702256

[129] Kanehisa, M., Furumichi, M., Sato, Y., Ishiguro-Watanabe, M. & Tanabe, M. KEGG: integrating viruses and cellular organisms. *Nucleic Acids Res.* **49**, D545–D551 (2021). 33125081 10.1093/nar/gkaa970 PMC7779016

[130] Love, M. I., Huber, W. & Anders, S. Moderated estimation of fold change and dispersion for RNA-seq data with DESeq2. *Genome Biol.* **15**, 1–21 (2014). 10.1186/s13059-014-0550-8 PMC4302049 25516281

