# Supplementary material for: Feeding the cosmos: tackling personalized space nutrition and the leaky gut challenge
Source: NPJ Microgravity. 2025 Jul 18;11:45. doi: 10.1038/s41526-025-00490-z (PMC12274595; doi:10.1038/s41526-025-00490-z)
Supplement: Supplementary file 1 — Supplementary material [file 41526_2025_490_MOESM1_ESM.pdf]

Supplementary Table 1

Nutrients	Type	NASA
Vitamin A	Fat-Soluble Vitamin	(RE = retinol equivalents; 1 RE = 1µg retinol or 6 µg ß-carotene) Vitamin A intake of preformed vitamin A (i.e., not from ß-carotene) should not exceed 3000 µg /d which is based on hepatotoxicity. Vitamin A is important for maintaining immune function, vision, and gene expression.
Vitamin D	Fat-Soluble Vitamin	This is designed to maintain vitamin D status in light of decreased exposure to ultraviolet light (and thus, decreased endogenous production). If not easily available in food sources, this shall be provided in supplement form. Vitamin D is required for maintaining serum calcium and phosphorus concentrations by influencing absorption of these nutrients in the small intestine. There is also evidence that vitamin D supports immune system function.
Vitamin K	Fat-Soluble Vitamin	Meeting this requirement is important because of the role of vitamin K in calcium and bone metabolism. Vitamin K requirement shall be provided as vitamin K-1 (phylloquinone), which is obtained primarily from plant sources.
Vitamin E	Fat-Soluble Vitamin	(TE = α-tocopherol equivalents; 1 TE = 1 mg d-α-tocopherol, which is the naturally sourced formed of vitamin E; synthetic forms have lower biological activity). Vitamin E serves as an antioxidant that prevents propagation of lipid peroxidation. Supplements should not contain any more than 1000 mg/d of any form of vitamin E based on the adverse effect of increased tendency to hemorrhage.
Vitamin C	Water-Soluble Vitamin	Vitamin C provides antioxidant protection, and this level of intake should maintain near maximal neutrophil vitamin C concentration with little urinary excretion. This intake level is 35 mg/d more than the current Dietary Reference Intake and is in agreement with their recommended intake for individuals exposed to increased oxidative stress (e.g., smokers). Intake from supplements and diet should not exceed 2000 mg/d based on the potential for osmotic diarrhea and gastrointestinal disturbances.
Vitamin B12	Water-Soluble Vitamin	Vitamin B12 is a cofactor for two enzymes involved in methyl transfer reactions ultimately involved with DNA synthesis.
Vitamin B6	Water-Soluble Vitamin	Vitamin B6 is a required coenzyme in the metabolism of amino acids and heme synthesis. Intake from supplements should not exceed 100 mg/d based on possible sensory neuropathy effects.
Thiamine	Water-Soluble Vitamin	Thiamine is a required coenzyme for carbohydrate and branched-chain amino acid metabolism.
Riboflavin	Water-Soluble Vitamin	Riboflavin is a required coenzyme for numerous redox reactions.
Folate	Water-Soluble Vitamin	Folate is required for DNA synthesis and methylation.
Niacin	Water-Soluble Vitamin	Niacin from supplements and food fortification should not exceed 35 mg/d based on flushing as a critical adverse effect. Niacin is required for energy utilization, redox reactions, biosynthetic reactions, and DNA replication and repair.
Biotin	Water-Soluble Vitamin	Biotin is a required nutrient for bicarbonate-dependent carboxylation reactions.
Pantothenic Acid	Water-Soluble Vitamin	Pantothenic acid is involved in fatty acid metabolism.
Choline	Water-Soluble Vitamin	Choline is a dietary component required for the structural integrity of cell membranes, neurotransmission, cell signaling, and lipid and cholesterol metabolism. Choline intake should not exceed 3.5 g/d related to concerns of cholinergic side effects.
Calcium	Mineral	Calcium is required to minimize bone and calcium imbalance during the mission. Calcium intake shall not exceed 2500 mg/d.
Phosphorus	Mineral	The phosphorus intake shall be 700 mg/d for men and women, and shall not exceed 1.5 times the calcium intake.
Magnesium	Mineral	The upper limit for both sexes is defined as 350 mg/d from supplements (i.e., not from dietary sources). Magnesium is a required cofactor for over 300 enzymes involved in energy production.
Sodium	Mineral	Sodium plays an important role in maintaining physiological homeostasis. Excess sodium has detrimental effects, including on bone metabolism and renal stone risk.
Potassium	Mineral	Potassium plays an important role in maintaining physiological homeostasis.
Iron	Mineral	Iron intake shall not exceed 8 mg/d for men and women. This is based on space-induced changes in iron storage, and is designed to prevent iron overload, a situation that may lead to oxidative tissue damage. Note: for women under 50 years of age who do not pharmacologically suppress menstruation, iron intake shall be at least 18 mg/d.
Copper	Mineral	Copper serves an important catalytic role in many metalloenzyme reactions to reduce molecular oxygen. These enzymes are involved with blocking histamine release, reactions to degrade serotonin, metabolism of catecholamines, and collagen cross-linking reactions. Intake of copper should not exceed 10 mg/d based on protection from liver damage.
Manganese	Mineral	Intake of manganese shall be 2.3 mg/d for men and 1.8 mg/d for women. Manganese is involved in the formation of bone and in lipid, carbohydrate, and amino acid metabolism. Intake should not exceed 11 mg/d.
Fluoride	Mineral	Fluoride is mainly associated with maintenance of calcified tissues.
Zinc	Mineral	Zinc serves many roles, including regulatory, structural, and catalytic functions. Intake should not exceed 40 mg/d, based on reduced red blood cell copper-zinc superoxide dismutase activity
Selenium	Mineral	Selenium is required for its role in oxidant defense, and it functions as a dietary antioxidant. Intake should not exceed 400 µg/d which is based on selenosis as the adverse effect.
Iodine	Mineral	Iodine is a required component of thyroid hormones. Intake should not exceed 1.1 mg/d, which is based on the effect on elevating serum thyroid stimulating hormone
Chromium	Mineral	Chromium is involved with maintaining glucose homeostasis
Chloride	Mineral	Chloride intake shall not exceed 3500 mg/d.
Molybdenum	Mineral	Molybdenum is involved in the catabolism of sulfur amino acids and heterocyclic compounds including purines and pyridines.

Supplementary Table 1. Nutrients description and needs regarding NASA astronaut guidelines

Supplementary Table 2

Plant (common name)	Species	Variety	Notes:
Red Romaine Lettuce	*Lactuca sativa*	“Outredgeous”	VEG-01 B (July 8, 2015 - Aug. 10, 2015), VEG-03 A (Oct. 25, 2016 – Dec. 28, 2016), Veg-03 F (Feb. 6, 2018 - April 6, 2018), Veg-03 E (Feb. 9, 2018 - April 9, 2018)
Green Romaine Lettuce	*Lactuca sativa*		
Mixed Lettuces	*Lactuca sativa*		
Dragoon' Lettuce	*Lactuca sativa*	"Dragoon"	VEG-03 G
‘Waldmann’s Green’ lettuce	*Lactuca sativa*	‘Waldmann’s Green’	VEG-03 D,E,F
Chinese cabbage	Brassica rapa	Tokyo Bekana	VEG-03 B,C
Mizuna mustard/lettuce	Brassica rapa	nipposinica	VEG-03 E,F
‘Extra Dwarf’ Pak Choi	Brassica rapa, chinensis	‘Extra Dwarf’ (a variety of chinensis)	VEG-03 H. This is the most compact Pak Choi available, yielding 2-inch tall, fully formed, wrinkled dark green leaves with thick white petioles. It matures in about 30 days and is suitable for salads, stir-fries, steamed dishes, and soups.
Red Russian' Kale	Brassica napus	‘Red Russian’	VEG-03 G
‘Wasabi’ Mustard Greens	Brassica juncea	‘Wasabi’	VEG-03 H. This variety is known for its spicy flavor, reminiscent of wasabi, making it a popular choice for adding a zesty kick to salads and dishes.
Wheat	Triticum aestivum		NASA experiments on space-grown dwarf wheat: Space Shuttle Columbia and ISS (1996–2003) (USU-Apogee Wheat), Salyut-7 Space Station (1982)
Rice	Oryza sativa		ISS Advanced Plant Habitat-01 (APH-01)
Potatos	Solanum tuberosum		Space Shuttle Columbia (STS-73) 1995. China’s Space Breeding Program onboard Shenzhou-14 2022. ISS.
Onions	Allium cepa		Salyut Space Stations (1971–1981). Cosmonauts conducted experiments in the “Oasis” greenhouse aboard the Salyut stations, successfully growing fresh green onions using hydroponic techniques. Tiangong Space Station (2023)
Peas	Pisum sativum		Salyut-7 Space Station (1982)
Radishes	Raphanus sativus		ISS APH Plant Habitat-02 (PH-02) 2020
Garlic	Allium sativum		Viktor Patsayev on Salyut 1 space station. 1971.
Cucumbers	Cucumis sativus		Japanese Aerospace Exploration Agency (JAXA) Studies 2011
Parsley	Petroselinum crispum		Salyut 6 Space Station (1977–1982). Cosmonaut Valery Ryumin cultivated a variety of plants, including parsley, using improvised containers such as empty film cassettes and food containers.
Dill	Anethum graveolens		Salyut 6 Space Station (1977–1982): Cosmonaut Valery Ryumin engaged in space gardening by repurposing items like empty film cassettes and food containers as plant pots.
Soybeans	*Glycine max*		ISS NASA Advanced Astroculture (ADVASC) Experiment (2002), Chinese University of Hong Kong (CUHK) Experiment on Tiangong Space Station (2023)
Flax	Linum usitatissimum		Viktor Patsayev on Salyut 1 space station. 1971.
Sunflower seeds	Helianthus annuus		Personal Experiment by Astronaut Don Pettit (2012)
Zucchini	Cucurbita pepo		Personal Experiment by Astronaut Don Pettit (2012). Expedition 30/31 on the ISS
Broccoli	Brassica oleracea		Personal Experiment by Astronaut Don Pettit (2012). ISS Probiotic-Coated Broccoli Seeds (2018)
Arabidopsis	Arabidopsis thaliana		APEX (many experiments). A model organism in plant biology and genetics due to its small genome, short life cycle, and ease of genetic manipulation.
Brachypodium	Brachypodium distachyon		APEX-06, 09. Commonly known as purple false brome, a model grass species in spaceflight experiments. Its small genome, short life cycle, and ease of genetic manipulation make it ideal for studying plant biology in microgravity.

Supplementary Table 2. Plant species and varieties tested on spaceflight experiments

Supplementary Table 3

Nutrient Details	Consequences
Calcium	Hypocalcemia, Osteoporosis, Rickets, Osteomalacia
Copper	Anemia, Neutropenia, Osteoporosis, Neurological symptoms
Fluoride	Dental Caries, Osteoporosis
Folate	Megaloblastic Anemia, Neural Tube Defects
Iodine	Goiter, Hypothyroidism, Cretinism
Iron	Iron Deficiency Anemia
Magnesium	Muscle Cramps, Osteoporosis, Hypertension
Riboflavin	Ariboflavinosis
Selenium	Keshan Disease, Kashin-Beck Disease, Weakened Immune System
Thiamine	Beriberi, Wernicke-Korsakoff Syndrome
Vitamin A	Night Blindness, Xerophthalmia, Increased Infection Risk
Vitamin B12	Pernicious Anemia, Neurological Disorders
Vitamin B6	Anemia, Dermatitis, Neurological Symptoms
Vitamin C	Scurvy
Vitamin D	Rickets, Osteomalacia, Osteoporosis
Vitamin E	Neurological Problems, Weakened Immune System
Zinc	Growth Retardation, Delayed Sexual Maturation, Impaired Immune Function, Skin Lesions

Supplementary Table 3. Diseases associated with nutrient imbalances on humans

Supplementary Table 5

Gene	p-value	Log2FC	Cell Type	Associated Process or Mineral	Study
PLCG2	0	2.1821512	B Cell	Calcium	I4
HLA-DRA	4.20E-118	-1.00101063	B Cell	Potassium	I4
CPED1	3.63E-70	0.61743681	B Cell	Iron	I4
CXCR4	1.06E-67	-0.73562154	B Cell	Calcium	I4
ITPR1	3.77E-49	0.56300662	B Cell	Calcium	I4
PTK2B	8.14E-49	0.46129902	B Cell	Calcium	I4
PRKCB	5.90E-44	0.4814461	B Cell	Calcium	I4
LMBRD1	2.25E-36	0.42044186	B Cell	Vitamin B12	I4
DRAM2	3.67E-36	0.3204344	B Cell	Potassium	I4
TPK1	8.10E-35	0.45495423	B Cell	Thaimine	I4
METTL15	3.62E-34	0.4348094	B Cell	Calcium	I4
SLC39A10	4.10E-32	0.41252487	B Cell	Zinc	I4
ITPR2	4.18E-22	0.33405855	B Cell	Calcium	I4
FTL	2.89E-19	-0.27459581	B Cell	Iron	I4
CCSER1	8.84E-15	0.5501852	B Cell	Copper	I4

Supplementary Table 5. Changes in expression of Calcium related genes in B cells from I4 astronauts

Supplementary Table 6

Supplementary Table 6. Changes in expression of Calcium related genes in different cell types and astronaut missions

Gene	CellType	Comparison	log2FoldChange	pvalue	qvalue	Mission	Nutrients (changes)	Diseases associated with the gene
PRDX5	CD16_Mono	FP4: R+1 vs L-44	-3.616	5.78E-10	0.0000177	I4	PRDX5 may mediate redox regulation of **Ca^{2+}** transporters at the ER-mitochondria interface, stabilizing calcium fluxes and preventing mitochondrial Ca^{2+} overload (source)	Alopecia Areata, Parkinson Disease 7, Autosomal Recessive Early-Onset
SLC25A6	CD14_Mono	FP4: R+1 vs L-44	-3.576	7.64E-101	2.34E-96	I4	Excessive mitochondrial matrix Ca2+ results in formation of the membrane transition pore (BAX, SLC25A6, ATP5), loss of membrane potential, release of cytochrome C and apoptosis (source)	Influenza, Bubonic Plague
PRDX5	CD14_Mono	FP4: R+1 vs L-44	-3.386	5.08E-33	1.55E-28	I4	PRDX5 may mediate redox regulation of **Ca^{2+}** transporters at the ER-mitochondria interface, stabilizing calcium fluxes and preventing mitochondrial Ca^{2+} overload (source)	Alopecia Areata, Parkinson Disease 7, Autosomal Recessive Early-Onset
SPR	CD4	Post-flight vs Pre-flight	-3.154	0.00101	0.00933	Twin		Dystonia, Dopa-Responsive, Due To Sepiapterin Reductase Deficiency and Dystonia
MPST	CD14_Mono	FP4: R+1 vs L-44	-3.135	1.78E-09	0.0000544	I4	Intracellular **calcium** levels were elevated after MPST siRNA treatment (source)	Mercaptolactate-Cysteine Disulfiduria and Cystathioninuria
MPST	CD14_Mono	FP4: R+1 vs L-44	-3.135	1.78E-09	0.0000544	I4	Intracellular **calcium** levels were elevated after MPST siRNA treatment (source)	Mercaptolactate-Cysteine Disulfiduria and Cystathioninuria
GPX4	CD14_Mono	FP4: R+1 vs L-44	-3.109	1.54E-40	4.70E-36	I4	**Selenium** utilization by GPX4 Is required to prevent hydroperoxide-induced ferroptosis (source)	Spondylometaphyseal Dysplasia, Sedaghatian Type, Bone Disease
GPX4	CD14_Mono	FP4: R+1 vs L-44	-3.109	1.54E-40	4.70E-36	I4	**Selenium** utilization by GPX4 Is required to prevent hydroperoxide-induced ferroptosis (source)	Spondylometaphyseal Dysplasia, Sedaghatian Type, Bone Disease
SHMT2	CD14_Mono	FP4: R+1 vs L-44	-2.971	3.35E-07	0.0103	I4	The absence of SHMT2 increased **serine** and **glycine** levels in circulation, decreased liver methylation potential, and increased susceptibility to fatty liver disease (source)	Neurodevelopmental Disorder With Cardiomyopathy, Spasticity, And Brain Abnormalities; Periventricular Heterotopia With Microcephaly, Autosomal Recessive
GPX4	CD16_Mono	FP4: R+1 vs L-44	-2.92	4.74E-09	0.000145	I4	**Selenium** utilization by GPX4 Is required to prevent hydroperoxide-induced ferroptosis (source)	Spondylometaphyseal Dysplasia, Sedaghatian Type, Bone Disease
GPX4	CD16_Mono	FP4: R+1 vs L-44	-2.92	4.74E-09	0.000145	I4	**Selenium** utilization by GPX4 Is required to prevent hydroperoxide-induced ferroptosis (source)	Spondylometaphyseal Dysplasia, Sedaghatian Type, Bone Disease
NAXE	CD14_Mono	FP4: R+1 vs L-44	-2.846	3.42E-07	0.0105	I4		Encephalopathy, Progressive, Early-Onset, With Brain Edema And/Or Leukoencephalopathy, 1; Nad Hx Epimerase Deficiency
SOD1	CD16_Mono	FP4: R+1 vs L-44	-2.773	7.65E-14	2.34E-09	I4	The protein encoded by the SOD1 gene binds **copper** and **zinc** ions and is one of two isozymes responsible for destroying free superoxide radicals in the body (source)	Spastic Tetraplegia And Axial Hypotonia, Progressive; Amyotrophic Lateral Sclerosis 1
SLC25A6	CD16_Mono	FP4: R+1 vs L-44	-2.755	2.51E-26	7.68E-22	I4	Excessive mitochondrial matrix Ca2+ results in formation of the membrane transition pore (BAX, SLC25A6, ATP5), loss of membrane potential, release of cytochrome C and apoptosis (source)	Influenza, Bubonic Plague
SLC25A6	DC	FP4: R+1 vs L-44	-2.72	9.74E-15	2.98E-10	I4	Excessive mitochondrial matrix Ca2+ results in formation of the membrane transition pore (BAX, SLC25A6, ATP5), loss of membrane potential, release of cytochrome C and apoptosis (source)	Influenza, Bubonic Plague
AKR7A2	CD16_Mono	LP2: R+82 vs all pre-flight	-2.676	3.82E-09	0.000117	I4		Gamma-Amino Butyric Acid Metabolism Disorder; Developmental Dysplasia Of The Hip 1
PRDX5	CD16_Mono	FP2: (R+1, R+45, R+82) vs all pre-flight	-2.591	2.58E-40	7.88E-36	I4		Alopecia Areata, Parkinson Disease 7, Autosomal Recessive Early-Onset
PRDX5	CD16_Mono	LP2: R+82 vs all pre-flight	-2.546	3.68E-35	1.13E-30	I4		Alopecia Areata, Parkinson Disease 7, Autosomal Recessive Early-Onset
ALAS2	CPT	In-flight, 1st Half vs Ground	-2.541	0.00000343	0.000512	Twin	ALAS2 catalyzes the first step of the heme biosynthetic pathway by condensing glycine and succinyl-CoA to form delta-aminolevulinic acid in the presence of pyridoxal 5'-phosphate, which is the **metabolite of vitamin B6** (source)	Anemia, Sideroblastic, 1; Protoporphyria, Erythropoietic, X-Linked
PRDX2	CD14_Mono	FP4: R+1 vs L-44	-2.506	8.56E-10	0.0000262	I4		Hemolytic Anemia, Alzheimer's Disease
NDUFAB1	CD14_Mono	FP4: R+1 vs L-44	-2.464	3.66E-21	1.12E-16	I4		Acute Gonococcal Prostatitis, Mitochondrial Disease
AKR7A2	CD16_Mono	FP2: (R+1, R+45, R+82) vs all pre-flight	-2.44	1.61E-09	0.0000494	I4		Gamma-Amino Butyric Acid Metabolism Disorder; Developmental Dysplasia Of The Hip 1
PRDX5	DC	FP2: (R+1, R+45, R+82) vs all pre-flight	-2.422	1.41E-32	4.31E-28	I4	PRDX5 may mediate redox regulation of **Ca^{2+}** transporters at the ER-mitochondria interface, stabilizing calcium fluxes and preventing mitochondrial Ca^{2+} overload (source)	Alopecia Areata, Parkinson Disease 7, Autosomal Recessive Early-Onset
PRDX5	DC	LP2: R+82 vs all pre-flight	-2.356	7.61E-29	2.33E-24	I4	PRDX5 may mediate redox regulation of **Ca^{2+}** transporters at the ER-mitochondria interface, stabilizing calcium fluxes and preventing mitochondrial Ca^{2+} overload (source)	Alopecia Areata, Parkinson Disease 7, Autosomal Recessive Early-Onset
ETHE1	CD14_Mono	FP4: R+1 vs L-44	-2.317	7.17E-12	2.19E-07	I4		Encephalopathy, Ethylmalonic; Succinic Semialdehyde Dehydrogenase Deficiency
ETFB	CD14_Mono	FP4: R+1 vs L-44	-2.161	3.53E-09	0.000108	I4		Multiple Acyl-Coa Dehydrogenase Deficiency; Multiple Acyl-Coa Dehydrogenase Deficiency, Mild Type
GPX4	other_T	FP4: R+1 vs L-44	-2.149	4.67E-07	0.0143	I4	**Selenium** utilization by GPX4 Is required to prevent hydroperoxide-induced ferroptosis (source)	Spondylometaphyseal Dysplasia, Sedaghatian Type, Bone Disease
GPX4	other_T	FP4: R+1 vs L-44	-2.149	4.67E-07	0.0143	I4	**Selenium** utilization by GPX4 Is required to prevent hydroperoxide-induced ferroptosis (source)	Spondylometaphyseal Dysplasia, Sedaghatian Type, Bone Disease
PRDX5	B	FP4: R+1 vs L-44	-2.119	4.70E-10	0.0000144	I4		Alopecia Areata and Parkinson Disease 7, Autosomal Recessive Early-Onset
SMDT1	CD16_Mono	FP2: (R+1, R+45, R+82) vs all pre-flight	-2.098	1.31E-20	4.02E-16	I4	Calcium (source)	Spastic Ataxia 5, Variola Mino
SMDT1	CD16_Mono	FP2: (R+1, R+45, R+82) vs all pre-flight	-2.098	1.31E-20	4.02E-16	I4	Calcium (source)	Spastic Ataxia 5, Variola Mino
SMDT1	CD16_Mono	FP2: (R+1, R+45, R+82) vs all pre-flight	-2.098	1.31E-20	4.02E-16	I4	Calcium (source)	Spastic Ataxia 5, Variola Mino
ETHE1	DC	FP2: (R+1, R+45, R+82) vs all pre-flight	-2.088	9.18E-13	2.81E-08	I4		Encephalopathy, Ethylmalonic; Succinic Semialdehyde Dehydrogenase Deficiency
SMDT1	CD14_Mono	FP4: R+1 vs L-44	-2.072	5.42E-10	0.0000166	I4	Calcium (source)	Spastic Ataxia 5, Variola Mino
SMDT1	CD14_Mono	FP4: R+1 vs L-44	-2.072	5.42E-10	0.0000166	I4	Calcium (source)	Spastic Ataxia 5, Variola Mino
SMDT1	CD14_Mono	FP4: R+1 vs L-44	-2.072	5.42E-10	0.0000166	I4	Calcium (source)	Spastic Ataxia 5, Variola Mino
SOD1	CD14_Mono	FP4: R+1 vs L-44	-2.054	1.53E-11	4.67E-07	I4		Spastic Tetraplegia And Axial Hypotonia, Progressive; Amyotrophic Lateral Sclerosis 1
TXN2	CD14_Mono	FP4: R+1 vs L-44	-2.048	1.34E-07	0.00411	I4		Combined Oxidative Phosphorylation Deficiency 29; Abdominal Obesity-Metabolic Syndrome 1
TXN2	CD14_Mono	FP4: R+1 vs L-44	-2.048	1.34E-07	0.00411	I4		Combined Oxidative Phosphorylation Deficiency 29; Abdominal Obesity-Metabolic Syndrome 1
ETHE1	DC	LP2: R+82 vs all pre-flight	-2.047	1.90E-11	5.81E-07	I4		Encephalopathy, Ethylmalonic; Succinic Semialdehyde Dehydrogenase Deficiency
SMDT1	CD16_Mono	LP2: R+82 vs all pre-flight	-2.031	9.86E-18	3.02E-13	I4	Calcium (source)	Spastic Ataxia 5, Variola Mino
SMDT1	CD16_Mono	LP2: R+82 vs all pre-flight	-2.031	9.86E-18	3.02E-13	I4	Calcium (source)	Spastic Ataxia 5, Variola Mino
SMDT1	CD16_Mono	LP2: R+82 vs all pre-flight	-2.031	9.86E-18	3.02E-13	I4	Calcium (source)	Spastic Ataxia 5, Variola Mino
GRPEL1	CPT	In-flight, 2nd Half vs Ground	-2.03	3.03E-08	5.12E-07	Twin		Autosomal Dominant Severe Congenital Neutropenia; Cystic Fibrosis
FTH1	CD16_Mono	FP4: R+1 vs L-44	-2.028	7.09E-44	2.17E-39	I4		Hemochromatosis, Type 5; Neurodegeneration With Brain Iron Accumulation 9
SLC25A3	CD16_Mono	FP4: R+1 vs L-44	-2.028	8.80E-15	2.69E-10	I4	Transports phosphate or copper ions across the mitochondrial inner membrane into the matrix compartment (source)	Mitochondrial Phosphate Carrier Deficiency, Cardiomyopathy-Hypotonia-Lactic Acidosis Syndrome
SLC25A3	CD16_Mono	FP4: R+1 vs L-44	-2.028	8.80E-15	2.69E-10	I4	Transports phosphate or copper ions across the mitochondrial inner membrane into the matrix compartment (source)	Mitochondrial Phosphate Carrier Deficiency, Cardiomyopathy-Hypotonia-Lactic Acidosis Syndrome
SLC25A3	CD16_Mono	FP4: R+1 vs L-44	-2.028	8.80E-15	2.69E-10	I4	Transports phosphate or copper ions across the mitochondrial inner membrane into the matrix compartment (source)	Mitochondrial Phosphate Carrier Deficiency, Cardiomyopathy-Hypotonia-Lactic Acidosis Syndrome
PRDX5	CD8_T	FP4: R+1 vs L-44	-2.015	1.27E-11	3.87E-07	I4		Alopecia Areata and Parkinson Disease 7, Autosomal Recessive Early-Onset
PRDX2	pbmc	FP4: R+1 vs L-44	-2	7.47E-100	2.29E-95	I4		Alopecia Areata and Parkinson Disease 7, Autosomal Recessive Early-Onset
AMT	CPT	In-flight, 2nd Half vs Ground	2.007	1.88E-20	5.69E-18	Twin		Glycine Encephalopathy 2; Glycine Encephalopathy
PPOX	CD14_Mono	LP1: R+45 vs all pre-flight	2.036	5.16E-11	0.00000158	I4		Variegate Porphyria; Variegate Porphyria, Childhood-Onset
ALDH2	CD4_T	LP1: R+45 vs all pre-flight	2.057	9.36E-10	0.0000286	I4		Alcohol Sensitivity, Acute; Amed Syndrome, Digenic
MCCC1	other	FP4: R+1 vs L-44	2.065	1.73E-07	0.00529	I4		3-Methylcrotonyl-Coa Carboxylase 1 Deficiency; 3-Methylcrotonyl-Coa Carboxylase Deficiency
IBA57	other	LP2: R+82 vs all pre-flight	2.066	7.75E-07	0.0237	I4		Multiple Mitochondrial Dysfunctions Syndrome 3; Spastic Paraplegia 74, Autosomal Recessive
ALDH1L2	CD8_T	FP4: R+1 vs L-44	2.077	1.96E-08	0.000599	I4		Sjogren-Larsson Syndrome, Succinic Semialdehyde Dehydrogenase Deficiency
ALDH2	pbmc	LP1: R+45 vs all pre-flight	2.084	5.73E-88	1.75E-83	I4		Alcohol Sensitivity, Acute; Amed Syndrome, Digenic
MGST1	CD4_T	LP2: R+82 vs all pre-flight	2.095	2.77E-20	8.49E-16	I4		Pulmonary Disease, Chronic Obstructive, Cataract
MGST1	CD4_T	LP2: R+82 vs all pre-flight	2.095	2.77E-20	8.49E-16	I4		Pulmonary Disease, Chronic Obstructive, Cataract
COX10	other	FP4: R+1 vs L-44	2.119	3.88E-07	0.0119	I4		Mitochondrial Complex Iii Deficiency, Nuclear Type 4; Cox Deficiency, Benign Infantile Mitochondrial Myopathy
KMO	pbmc	FP4: R+1 vs L-44	2.136	1.17E-181	3.57E-177	I4		Transient Cerebral Ischemia, Streptococcal Meningitis
NOCT	B	LP1: R+45 vs all pre-flight	2.141	3.07E-11	9.39E-07	I4		
MGST1	CD4_T	FP2: (R+1, R+45, R+82) vs all pre-flight	2.165	8.71E-23	2.67E-18	I4	Its main function is the conjugation of reduced glutathione to a wide number of exogenous and endogenous hydrophobic electrophiles (source)	Pulmonary Disease, Chronic Obstructive, Cataract
MGST1	CD4_T	FP2: (R+1, R+45, R+82) vs all pre-flight	2.165	8.71E-23	2.67E-18	I4		Pulmonary Disease, Chronic Obstructive, Cataract
SLC25A37	other	FP4: R+1 vs L-44	2.263	4.78E-10	0.0000146	I4		Anemia, Sideroblastic, 1; Protoporphyria, Erythropoietic, 1
NOCT	NK	LP1: R+45 vs all pre-flight	2.275	8.44E-13	2.58E-08	I4		
ALDH2	pbmc	LP2: R+82 vs all pre-flight	2.315	0	0	I4		Alcohol Sensitivity, Acute; Amed Syndrome, Digenic
ALDH2	pbmc	FP2: (R+1, R+45, R+82) vs all pre-flight	2.367	0	0	I4		Alcohol Sensitivity, Acute; Amed Syndrome, Digenic
RHOT2	CPT	In-flight, 2nd Half vs Ground	2.394	3.32E-10	9.32E-09	Twin		Neuropathy, Congenital Hypomyelinating, 1, Autosomal Recessive and 3-Methylglutaconic Aciduria, Type Iii
ALDH2	NK	LP2: R+82 vs all pre-flight	2.396	5.08E-17	1.56E-12	I4		Alcohol Sensitivity, Acute; Amed Syndrome, Digenic
ALDH2	NK	FP2: (R+1, R+45, R+82) vs all pre-flight	2.429	9.59E-19	2.93E-14	I4		Alcohol Sensitivity, Acute; Amed Syndrome, Digenic
ALDH2	other_T	FP2: (R+1, R+45, R+82) vs all pre-flight	2.451	1.34E-19	4.10E-15	I4		Alcohol Sensitivity, Acute; Amed Syndrome, Digenic
ALDH2	other_T	LP2: R+82 vs all pre-flight	2.524	8.07E-21	2.47E-16	I4		Alcohol Sensitivity, Acute; Amed Syndrome, Digenic
MGST1	pbmc	LP2: R+82 vs all pre-flight	2.638	4.11E-300	1.26E-295	I4	Its main function is the conjugation of reduced glutathione to a wide number of exogenous and endogenous hydrophobic electrophiles (source)	Pulmonary Disease, Chronic Obstructive, Cataract
MGST1	pbmc	LP2: R+82 vs all pre-flight	2.638	4.11E-300	1.26E-295	I4		Pulmonary Disease, Chronic Obstructive, Cataract
MGST1	CD8_T	FP2: (R+1, R+45, R+82) vs all pre-flight	2.657	4.93E-11	0.00000151	I4		Pulmonary Disease, Chronic Obstructive, Cataract
MGST1	CD8_T	FP2: (R+1, R+45, R+82) vs all pre-flight	2.657	4.93E-11	0.00000151	I4		Pulmonary Disease, Chronic Obstructive, Cataract
PPIF	pbmc	LP1: R+45 vs all pre-flight	2.664	1.07E-178	3.29E-174	I4		Ullrich Congenital Muscular Dystrophy 1A, Alzheimer's Disease
MGST1	pbmc	FP2: (R+1, R+45, R+82) vs all pre-flight	2.686	0	0	I4		Pulmonary Disease, Chronic Obstructive and Cataract
MGST1	pbmc	FP2: (R+1, R+45, R+82) vs all pre-flight	2.686	0	0	I4		Pulmonary Disease, Chronic Obstructive and Cataract
GLYAT	pbmc	FP4: R+1 vs L-44	2.726	5.66E-10	0.0000173	I4	It detoxifies xenobiotics, such as benzoic acid or salicylic acid, and endogenous organic acids, such as isovaleric acid (source)	Glutaric Aciduria Iii and Osteogenesis Imperfecta, Type I
MGST1	CD8_T	LP2: R+82 vs all pre-flight	2.782	6.74E-12	2.06E-07	I4		Pulmonary Disease, Chronic Obstructive and Cataract
MGST1	CD8_T	LP2: R+82 vs all pre-flight	2.782	6.74E-12	2.06E-07	I4		Pulmonary Disease, Chronic Obstructive and Cataract
QDPR	other	FP4: R+1 vs L-44	2.815	4.47E-08	0.00137	I4		Hyperphenylalaninemia, Bh4-Deficient, C and Hyperphenylalaninemia, Bh4-Deficient, A
QDPR	other	FP4: R+1 vs L-44	2.815	4.47E-08	0.00137	I4		Hyperphenylalaninemia, Bh4-Deficient, C and Hyperphenylalaninemia, Bh4-Deficient, A
MPST	CPT	In-flight, 2nd Half vs Ground	2.947	3.07E-12	1.48E-10	Twin		Mercaptolactate-Cysteine Disulfiduria and Cystathioninuria
MPST	CPT	In-flight, 2nd Half vs Ground	2.947	3.07E-12	1.48E-10	Twin		Mercaptolactate-Cysteine Disulfiduria and Cystathioninuria
MGST1	CD4_T	LP1: R+45 vs all pre-flight	3.02	2.46E-10	0.00000751	I4	Its main function is the conjugation of reduced glutathione to a wide number of exogenous and endogenous hydrophobic electrophiles (source)	Pulmonary Disease, Chronic Obstructive and Cataract
MGST1	CD4_T	LP1: R+45 vs all pre-flight	3.02	2.46E-10	0.00000751	I4		Pulmonary Disease, Chronic Obstructive and Cataract
MGST1	B	FP2: (R+1, R+45, R+82) vs all pre-flight	3.106	2.56E-09	0.0000783	I4		Pulmonary Disease, Chronic Obstructive and Cataract
MGST1	B	FP2: (R+1, R+45, R+82) vs all pre-flight	3.106	2.56E-09	0.0000783	I4		Pulmonary Disease, Chronic Obstructive and Cataract
MGST1	B	LP2: R+82 vs all pre-flight	3.145	6.26E-09	0.000191	I4		Pulmonary Disease, Chronic Obstructive and Cataract
MGST1	B	LP2: R+82 vs all pre-flight	3.145	6.26E-09	0.000191	I4		Pulmonary Disease, Chronic Obstructive and Cataract
ALDH2	pbmc	FP4: R+1 vs L-44	3.267	0	0	I4		Pulmonary Disease, Chronic Obstructive and Cataract
MGST1	CD4_T	FP4: R+1 vs L-44	3.684	1.60E-13	4.90E-09	I4		Pulmonary Disease, Chronic Obstructive and Cataract
MGST1	CD4_T	FP4: R+1 vs L-44	3.684	1.60E-13	4.90E-09	I4		Pulmonary Disease, Chronic Obstructive and Cataract
MGST1	B	FP4: R+1 vs L-44	3.862	2.39E-07	0.0073	I4		Pulmonary Disease, Chronic Obstructive and Cataract
MGST1	B	FP4: R+1 vs L-44	3.862	2.39E-07	0.0073	I4		Pulmonary Disease, Chronic Obstructive and Cataract
MGST1	pbmc	FP4: R+1 vs L-44	4.081	4.29E-229	1.31E-224	I4		Pulmonary Disease, Chronic Obstructive and Cataract
MGST1	pbmc	FP4: R+1 vs L-44	4.081	4.29E-229	1.31E-224	I4		Pulmonary Disease, Chronic Obstructive and Cataract
ALDH7A1	CD16_Mono	LP1: R+45 vs all pre-flight	4.488	0.00000114	0.0348	I4	Vitamin B6	Epilepsy, Early-Onset, 4, Vitamin B6-Dependent, Galactosemia Iii
ALDH7A1	CD16_Mono	LP1: R+45 vs all pre-flight	4.488	0.00000114	0.0348	I4	Vitamin B6	Epilepsy, Early-Onset, 4, Vitamin B6-Dependent, Galactosemia Iii
ALDH7A1	CD16_Mono	LP1: R+45 vs all pre-flight	4.488	0.00000114	0.0348	I4	Vitamin B6	Epilepsy, Early-Onset, 4, Vitamin B6-Dependent, Galactosemia Iii
SPR	other_T	LP1: R+45 vs all pre-flight	5.803	2.72E-07	0.00832	I4		Dystonia, Dopa-Responsive, Due To Sepiapterin Reductase Deficiency and Dystonia
ACSM2A	NK	LP1: R+45 vs all pre-flight	5.87	1.26E-07	0.00386	I4		Spherocytosis, Type 3; Abdominal Obesity-Metabolic Syndrome 1
ACSM5	CD8_T	LP1: R+45 vs all pre-flight	5.886	9.01E-08	0.00276	I4		X-Linked Epilepsy With Variable Learning Disabilities, Behavior Disorders, Abdominal Obesity-Metabolic Syndrome 1
ALDH1L1	DC	LP1: R+45 vs all pre-flight	6.327	2.44E-10	0.00000746	I4		Focal Dermal Hypoplasia and Intellectual Developmental Disorder, Autosomal Recessive 59

Supplementary Table 7

Supplementary Table 7: Nutrient related gene variants and their associated diseases and drugs.

Disease	Nutrient/Mine	Drug	Gene	Variant	Therapeutic Area	Phenotype
Cancer	Aluminum	cisplatin	GSTA1	rs3957356	Other specified gliomas of brain; Other specified malignant neoplasms of the ovary; Other specified malignant neoplasms of bronchus or lung; [illegible]	
Cancer	Aluminum	dacarbazine	GSTM1	rs3754446	Hodgkin lymphoma	
Cancer	Aluminum	oxaliplatin	GSTM1	rs3754446	Malignant neoplasm metastasis in large intestine; Malignant neoplasms of colon	
Cancer	Aluminum	vinblastine	GSTM1	rs3754446	Other specified gliomas of brain; Kaposi sarcoma of unspecified primary site; Hodgkin lymphoma; Other specified malignant neoplasms of the ovary; [illegible]	
Cancer	Aluminum	vincristine	GSTA1	rs3957357	Other specified gliomas of brain; Malignant trophoblastic neoplasms of placenta; Follicular lymphoma; Kaposi sarcoma of unspecified primary site; [illegible]	Lymphoma, Large
Cancer	Aluminum	doxorubicin	GSTM1	rs3754446	Malignant neoplasms of breast; Hodgkin lymphoma; Diffuse large B-cell lymphomas; Plasma cell myeloma; Follicular lymphoma; Burkitt lymphoma [illegible]	
Cancer	Aluminum	prednisone	GSTA1	rs3957357	Plasma cell myeloma; Follicular lymphoma; Lymphoid leukaemia, not elsewhere classified; Malignant neoplasms of prostate; Chronic lymphocytic [illegible]	Lymphoma, Large
	Aluminum	clozapine	GSTM1	rs3754446	Schizophrenia or other primary psychotic disorders	
Cancer	Calcium	capecitabine	VEGFA	rs3025040	Other specified malignant neoplasms of breast; Malignant neoplasm metastasis in large intestine; Malignant neoplasms of rectum; Malignant [illegible]	
Cancer	Calcium	carboplatin	ERBB3	rs2229046	Other specified gliomas of brain; Other specified carcinomas of ovary; Retinoblastoma; Other specified malignant neoplasms of bronchus or lung; [illegible]	Breast Neoplasms
Cancer	Calcium	carboplatin	VEGFA	rs3025040	Other specified gliomas of brain; Other specified carcinomas of ovary; Retinoblastoma; Other specified malignant neoplasms of bronchus or lung; [illegible]	
Cancer	Calcium	cisplatin	VEGFA	rs833069	Other specified gliomas of brain; Other specified malignant neoplasms of the ovary; Other specified malignant neoplasms of bronchus or lung; [illegible]	
Cancer	Calcium	cisplatin	VEGFA	rs1320735	Other specified gliomas of brain; Other specified malignant neoplasms of the ovary; Other specified malignant neoplasms of bronchus or lung; [illegible]	
Cancer	Calcium	cyclophosphami	VEGFA	rs833069	Other specified malignant neoplasms of breast; Malignant trophoblastic neoplasms of placenta; Follicular lymphoma; Malignant neoplasms of breast; [illegible]	
Cancer	Calcium	cyclophosphami	VEGFA	rs1320735	Other specified malignant neoplasms of breast; Malignant trophoblastic neoplasms of placenta; Follicular lymphoma; Malignant neoplasms of breast; [illegible]	
Cancer	Calcium	docetaxel	VEGFA	rs3025040	Other specified malignant neoplasms of breast; Malignant neoplasms of breast; Malignant neoplasms of prostate	
Cancer	Calcium	irinotecan	VEGFA	rs3025040	Malignant neoplasm metastasis in large intestine; Malignant neoplasms of kidney, except renal pelvis; Rhabdomyosarcoma primary site	
Cancer	Calcium	methotrexate	ADORA2A	rs3761422	Lymphoid leukaemia, not elsewhere classified; Acute myeloid leukaemia with recurrent genetic abnormalities; Malignant trophoblastic neoplasms of [illegible]	Arthritis,
Cancer	Calcium	oxaliplatin	VEGFA	rs3025040	Malignant neoplasm metastasis in large intestine; Malignant neoplasms of colon	
Cancer	Calcium	regorafenib	KDR	rs4864950	Oncology	Drug Toxicity
Cancer	Calcium	trastuzumab	ERBB3	rs2229046	Carcinoma of breast, specialised type	Breast Neoplasms
Cancer	Calcium	trastuzumab	ERBB2	rs1136201	Carcinoma of breast, specialised type	Breast Neoplasms
Cancer	Calcium	daunorubicin	NOS3	rs1799983	Acute myeloid leukaemia with recurrent genetic abnormalities; Lymphoid leukaemia, not elsewhere classified; Myeloid leukaemia	Leukemia, Myeloid,
Hypertension	Calcium	atenolol	ADRB2	rs1042714	Essential hypertension	Tachycardia
Hypertension	Calcium	atenolol	PRKCB	rs9922316	Essential hypertension	
Hypertension	Calcium	atenolol	ADRB2	rs1042718	Essential hypertension	
Hypertension	Calcium	hydrochlorothia	PRKCA	rs4791040	Essential hypertension; Heart failure; Oedema	Hypertension
Hypertension	Calcium	verapamil	NOS1AP	rs1091859	Supraventricular tachyarrhythmia; Angina pectoris	Acquired Long QT
Hypertension	Calcium	verapamil	NOS1AP	rs1049436	Supraventricular tachyarrhythmia; Angina pectoris	Acquired Long QT
Hypertension	Calcium	verapamil	CACNA1A	rs2112460	Supraventricular tachyarrhythmia; Angina pectoris	
Hypertension	Calcium	metoprolol	ADRB2	rs1042718	Angina pectoris; Heart failure; Essential hypertension; Cardiac arrhythmia	
	Calcium	clozapine	NTRK2	rs1778929	Schizophrenia or other primary psychotic disorders	Schizophrenia
	Calcium	clozapine	HTR7	rs1935349	Schizophrenia or other primary psychotic disorders	
	Calcium	fluorouracil	VEGFA	rs1320735	Plantar warts; Malignant neoplasms of nasopharynx; Malignant neoplasm metastasis in large intestine; Malignant neoplasms of breast; Malignant [illegible]	
	Calcium	fluorouracil	VEGFA	rs833069	Plantar warts; Malignant neoplasms of nasopharynx; Malignant neoplasm metastasis in large intestine; Malignant neoplasms of breast; Malignant [illegible]	
	Calcium	pravastatin	HTR7	rs1935349	Mixed hyperlipidaemia; Coronary atherosclerosis	Myalgia
Miscellaneou	Calcium /	calcium	VDR	rs731236	Calcium deficiency	
Cancer	Copper	carboplatin	SLC31A1	rs1081746	Other specified gliomas of brain; Other specified carcinomas of ovary; Retinoblastoma; Other specified malignant neoplasms of bronchus or lung; [illegible]	
Cancer	Copper	carboplatin	SLC31A1	rs4978536	Other specified gliomas of brain; Other specified carcinomas of ovary; Retinoblastoma; Other specified malignant neoplasms of bronchus or lung; [illegible]	
Cancer	Copper	cisplatin	SLC31A1	rs2233914	Other specified gliomas of brain; Other specified malignant neoplasms of the ovary; Other specified malignant neoplasms of bronchus or lung; [illegible]	
Cancer	Folate	carboplatin	SLC19A1	rs914232	Other specified gliomas of brain; Other specified carcinomas of ovary; Retinoblastoma; Other specified malignant neoplasms of bronchus or lung; [illegible]	
Cancer	Folate	carboplatin	MTHFR	rs1742151	Other specified gliomas of brain; Other specified carcinomas of ovary; Retinoblastoma; Other specified malignant neoplasms of bronchus or lung; [illegible]	
Cancer	Folate	cisplatin	MTHFD1	rs2236225	Other specified gliomas of brain; Other specified malignant neoplasms of the ovary; Other specified malignant neoplasms of bronchus or lung; [illegible]	Osteosarcoma
Cancer	Folate	cisplatin	SLC19A1	rs12659	Other specified gliomas of brain; Other specified malignant neoplasms of the ovary; Other specified malignant neoplasms of bronchus or lung; [illegible]	Uterine Cervical
Cancer	Folate	cisplatin	MTHFR	rs1801131	Other specified gliomas of brain; Other specified malignant neoplasms of the ovary; Other specified malignant neoplasms of bronchus or lung; [illegible]	Neoplasms
Cancer	Folate	cisplatin	MTHFR	rs4846051	Other specified gliomas of brain; Other specified malignant neoplasms of the ovary; Other specified malignant neoplasms of bronchus or lung; [illegible]	
Cancer	Folate	cisplatin	SLC19A1	rs1051298	Other specified gliomas of brain; Other specified malignant neoplasms of the ovary; Other specified malignant neoplasms of bronchus or lung; [illegible]	
Cancer	Folate	cisplatin	SLC19A1	rs2838958	Other specified gliomas of brain; Other specified malignant neoplasms of the ovary; Other specified malignant neoplasms of bronchus or lung; [illegible]	
Cancer	Folate	imatinib	SLC19A1	rs914232	B lymphoblastic leukaemia or lymphoma with t(9:22) (q34;q11.2); BCR-ABL1; Gastrointestinal stromal tumour of unspecified gastrointestinal sites; [illegible]	
Cancer	Folate	irinotecan	SLC19A1	rs914232	Malignant neoplasm metastasis in large intestine; Malignant neoplasms of kidney, except renal pelvis; Rhabdomyosarcoma primary site	
Cancer	Folate	mercaptopurine	MTHFR	rs1742151	Acute myeloid leukaemia with recurrent genetic abnormalities; Lymphoid leukaemia, not elsewhere classified; Langerhans cell histiocytosis	
Cancer	Folate	methotrexate	MTHFR	rs4846051	Lymphoid leukaemia, not elsewhere classified; Acute myeloid leukaemia with recurrent genetic abnormalities; Malignant trophoblastic neoplasms of [illegible]	Arthritis,
Cancer	Folate	methotrexate	MTHFD1	rs2236225	Lymphoid leukaemia, not elsewhere classified; Acute myeloid leukaemia with recurrent genetic abnormalities; Malignant trophoblastic neoplasms of [illegible]	Anemia,Leukopeni
Cancer	Folate	methotrexate	DHFR	rs1650723	Lymphoid leukaemia, not elsewhere classified; Acute myeloid leukaemia with recurrent genetic abnormalities; Malignant trophoblastic neoplasms of [illegible]	Osteosarcoma
Cancer	Folate	methotrexate	MTHFR	rs1801131	Lymphoid leukaemia, not elsewhere classified; Acute myeloid leukaemia with recurrent genetic abnormalities; Malignant trophoblastic neoplasms of [illegible]	Burkitt
Cancer	Folate	methotrexate	SLC19A1	rs12659	Lymphoid leukaemia, not elsewhere classified; Acute myeloid leukaemia with recurrent genetic abnormalities; Malignant trophoblastic neoplasms of [illegible]	
Cancer	Folate	methotrexate	SLC19A1	rs1051298	Lymphoid leukaemia, not elsewhere classified; Acute myeloid leukaemia with recurrent genetic abnormalities; Malignant trophoblastic neoplasms of [illegible]	
Cancer	Folate	oxaliplatin	MTHFR	rs1742151	Malignant neoplasm metastasis in large intestine; Malignant neoplasms of colon	
Cancer	Folate	doxorubicin	MTHFR	rs1742151	Malignant neoplasms of breast; Hodgkin lymphoma; Diffuse large B-cell lymphomas; Plasma cell myeloma; Follicular lymphoma; Burkitt lymphoma [illegible]	
Miscellaneou	Folate	folic acid	MTHFR	rs1801131	Folate deficiency anaemia; Anencephaly or similar anomalies; Spina bifida; Cephalocele	Neoplasms
Miscellaneou	Folate	folic acid	MTHFR	rs1801133	Folate deficiency anaemia; Anencephaly or similar anomalies; Spina bifida; Cephalocele	
Miscellaneous	Folate	folic acid	MTHFR	rs17421511	Folate deficiency anaemia; Anencephaly or similar anomalies; Spina bifida; Cephalocele	
	Folate	clozapine	MTHFR	rs17421511	Schizophrenia or other primary psychotic disorders	
	Folate	fluorouracil	SLC19A1	rs12659	Plantar warts; Malignant neoplasms of nasopharynx; Malignant neoplasm metastasis in large intestine; Malignant neoplasms of breast; Malignant neoplasms of rectum; Malignant neoplasms of colon	Uterine Cervical Neoplasms
	Folate	fluorouracil	MTHFR	rs1801131	Plantar warts; Malignant neoplasms of nasopharynx; Malignant neoplasm metastasis in large intestine; Malignant neoplasms of breast; Malignant neoplasms of rectum; Malignant neoplasms of colon	Neoplasms
	Folate	fluorouracil	MTHFR	rs4846051	Plantar warts; Malignant neoplasms of nasopharynx; Malignant neoplasm metastasis in large intestine; Malignant neoplasms of breast; Malignant neoplasms of rectum; Malignant neoplasms of colon	
	Folate	pravastatin	MTHFR	rs17421511	Mixed hyperlipidaemia; Coronary atherosclerosis	
	Folate	nitrous oxide	MTHFR	rs1801131	Anaesthetics and therapeutic gases	
Cancer	Magnesium	dacomitinib	EGFR	rs11506105	Oncology	
Hypertension	Potassium	atenolol	SLC4A1	rs45545233	Essential hypertension	Hypertension
Hypertension	Potassium / Sodium	hydrochlorothiazide	SLC12A3	rs1529927	Essential hypertension; Heart failure; Oedema	
Miscellaneou	Vitamin B12	folic acid	MTRR	rs1801394	Folate deficiency anaemia; Anencephaly or similar anomalies; Spina bifida; Cephalocele	Migraine with Aura
Cancer	Vitamin K	cisplatin	EPHX1	rs2234922	Other specified gliomas of brain; Other specified malignant neoplasms of the ovary; Other specified malignant neoplasms of bronchus or lung; Malignant neoplasms of nasopharynx; Germ cell tumour of testis; Osteosarcoma of bone and articular cartilage of unspecified sites; Malignant neoplasms of lip, oral cavity or pharynx; Malignant neoplasms of cervix uteri	
Cancer	Vitamin K	cyclophosphamide	EPHX1	rs2234922	Other specified malignant neoplasms of breast; Malignant trophoblastic neoplasms of placenta; Follicular lymphoma; Malignant neoplasms of breast; Chronic lymphocytic leukaemia or small lymphocytic lymphoma; Rhabdomyosarcoma primary site; Hodgkin lymphoma; Diffuse large B-cell lymphomas; Plasma cell myeloma; Other specified gliomas of brain; Burkitt lymphoma including Burkitt leukaemia; Malignant neoplasms of kidney, except renal pelvis; Ewing sarcoma of bone and articular cartilage of unspecified sites; Lymphoid leukaemia, not elsewhere classified; Anaplastic large cell lymphoma, ALK-negative; Anaplastic large cell lymphoma, ALK-positive	
Hypertension	Vitamin K	warfarin	VKORC1	rs2884737	Other specified diseases of arteries or arterioles; Cerebral ischaemic stroke due to embolic occlusion; Venous thromboembolism; Atrial fibrillation	
Hypertension	Vitamin K	warfarin	VKORC1	rs61162043	Other specified diseases of arteries or arterioles; Cerebral ischaemic stroke due to embolic occlusion; Venous thromboembolism; Atrial fibrillation	
Hypertension	Vitamin K	warfarin	VKORC1	rs9923231	Other specified diseases of arteries or arterioles; Cerebral ischaemic stroke due to embolic occlusion; Venous thromboembolism; Atrial fibrillation	Hemorrhage

Supplementary Table 8

OSD	Reference	Purpose	Organism
https://osdr.nasa.gov/bio/repo/data/studies/OSD-269	https://osdr.nasa.gov/bio/repo/data/studies/OSD-269	Nutritional analysis of lettuce grown on the ISS	Plant
https://osdr.nasa.gov/bio/repo/data/studies/OSD-268	https://osdr.nasa.gov/bio/repo/data/studies/OSD-268		
https://osdr.nasa.gov/bio/repo/data/studies/OSD-267	https://osdr.nasa.gov/bio/repo/data/studies/OSD-267		
OSD-745 (in curation)	Khodadad CLM, Hummerick ME, Spencer LE, Dixit AR, Richards JT, Romeyn MW, Smith TM, Wheeler RM, Massa GD. Microbiological and Nutritional Analysis of Lettuce Crops Grown on the International Space Station. Front Plant Sci. 2020 Mar 6;11:199. doi: 10.3389/fpls.2020.00199. PMID: 32210992; PMCID: PMC7067979.		
N/A	Shen, Y., Guo, S., Zhao, P., Wang, L., Wang, X., Li, J., et al. (2018). Research on Lettuce Growth Technology Onboard Chinese Tiangong II Spacelab. *Acta Astronautica* 144, 97–102. doi:10.1016/j.actaastro.2017.11.007	Nutritional analysis of lettuce grown on Tiangong II	
OSD-796	Dmitry Kurouski, Axell Rodriguez, Borja Barbero Barcenilla et al. Raman Spectroscopy as a Tool for Assessing Plant Growth in Space and on Lunar Regolith Simulants, 28 August 2024, PREPRINT (Version 1) available at Research Square [https://doi.org/10.21203/rs.3.rs-4801715/v1]	Secondary metabolites of Arabidopsis thaliana flown at the ISS	
https://osdr.nasa.gov/bio/repo/data/studies/OSD-569<br>https://osdr.nasa.gov/bio/repo/data/studies/OSD-570<br>https://osdr.nasa.gov/bio/repo/data/studies/OSD-571<br>https://osdr.nasa.gov/bio/repo/data/studies/OSD-572<br>https://osdr.nasa.gov/bio/repo/data/studies/OSD-573<br>https://osdr.nasa.gov/bio/repo/data/studies/OSD-574<br>https://osdr.nasa.gov/bio/repo/data/studies/OSD-575<br>https://osdr.nasa.gov/bio/repo/data/studies/OSD-630<br>https://osdr.nasa.gov/bio/repo/data/studies/OSD-656<br>https://osdr.nasa.gov/bio/repo/data/studies/OSD-687	Overbey, E.G., Kim, J., Tierney, B.T. *et al.* The Space Omics and Medical Atlas (SOMA) and international astronaut biobank. *Nature* **632**, 1145–1154 (2024). https://doi.org/10.1038/s41586-024-07639-y	Analysis of human health data from Inspiration 4	Human
	Jones, C.W., Overbey, E.G., Lacombe, J. *et al.* Molecular and physiological changes in the SpaceX Inspiration4 civilian crew. *Nature* **632**, 1155–1164 (2024). https://doi.org/10.1038/s41586-024-07648-x		
https://osdr.nasa.gov/bio/repo/data/studies/OSD-530<br>https://osdr.nasa.gov/bio/repo/data/studies/OSD-570<br>https://osdr.nasa.gov/bio/repo/data/studies/OSD-575	Kim, J., Tierney, B.T., Overbey, E.G. *et al.* Single-cell multi-ome and immune profiles of the Inspiration4 crew reveal conserved, cell-type, and sex-specific responses to spaceflight. *Nat Commun* **15**, 4954 (2024). https://doi.org/10.1038/s41467-024-49211-2		
https://osdr.nasa.gov/bio/repo/data/studies/OSD-569	Grigorev, K., Nelson, T.M., Overbey, E.G. *et al.* Direct RNA sequencing of astronaut blood reveals spaceflight-associated m6A increases and hematopoietic transcriptional responses. *Nat Commun* **15**, 4950 (2024). https://doi.org/10.1038/s41467-024-48929-3		
N/A	Overbey, E.G., Ryon, K., Kim, J. *et al.* Collection of biospecimens from the inspiration4 mission establishes the standards for the space omics and medical atlas (SOMA). *Nat Commun* **15**, 4964 (2024). https://doi.org/10.1038/s41467-024-48806-z		
N/A	Francine E. Garrett-Bakelman *et al.* The NASA Twins Study: A multidimensional analysis of a year-long human spaceflight.Science364,eaau8650(2019).DOI:10.1126/science.aau8650	Analysis of human health data from Twin study	
N/A	https://www.cell.com/c/the-biology-of-spaceflight		
https://osdr.nasa.gov/bio/repo/data/studies/OSD-530	https://osdr.nasa.gov/bio/repo/data/studies/OSD-530	Analysis of human health data from JAXA Cell-Free Epigenome analysis	

Supplementary Table 8. Database sources used for study.

Supplementary Figure 1

A.

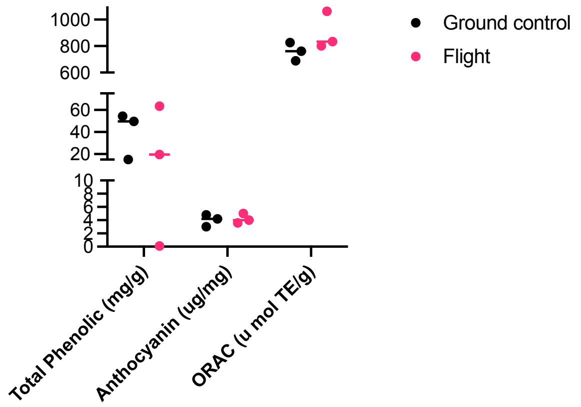

B.

Space environment	Study	Secondary metabolites	Recommended intake	Ground	Flight	Diseases associated with low intake
ISS veggie	Khodadad et al, 2020	Total Phenolic (mg/g)	450	39.67	27.70	Cardiovascular diseases, diabetes mellitus, and cancer
		Anthocyanin (ug/mg)	12.5	4.00	4.20	Cardiovascular diseases (including heart disease, hypertension, and stroke). Type 2 diabetes. Liver diseases. Osteoporosis, Age-related macular degeneration. Oxidative stress related conditions
		ORAC (u mol TE/g)	NA	758.77	900.07	NA

Supplementary Figure 1. Total Phenolics, anthocyanin and ORAC measurements for lettuce grown aboard the ISS

Supplementary Figure 2

A.

I4 | JAXA | Twin

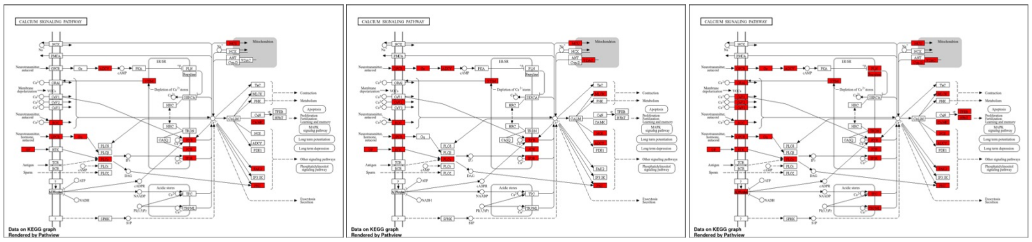

B.

Feature	I4	JAXA	Twin
Intracellular	Strong ER focus, limited mitochondrial	Balanced ER and mitochondrial signaling	ER, mitochondrial, and nuclear signaling
Extracellular	Minimal	Moderate (VGCCs active)	Extensive
Bone Health	Impaired osteoblast activity	Osteoclast activation	Widespread disruption
Oxidative Stress	Low	Moderate	High
Muscle Atrophy	Indirect	Moderate	Severe

Supplementary Figure 2. KEGG analysis for calcium signaling pathways for I4, JAXA and Twin study missions

Supplementary Figure 3

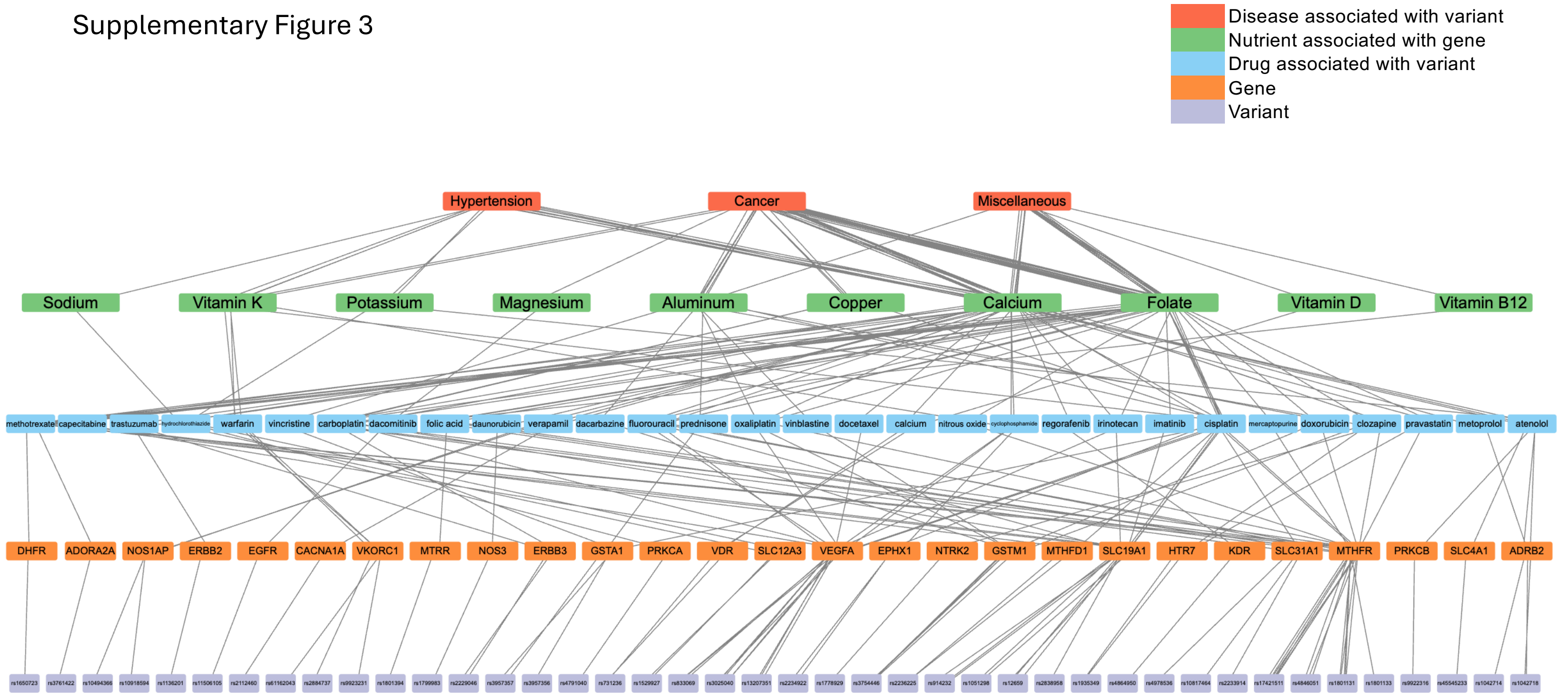

Supplementary Figure 3. Diseases, nutrients and drugs associated with gene variants observed in I4 mission
